# Carbene transfer reactivity from a nickelacyclobutane†

**DOI:** 10.1039/d4cc04273e

**Published:** 2024-09-30

**Authors:** María L. G. Sansores-Paredes, Martin Lutz, Marc-Etienne Moret

**Affiliations:** a Organic Chemistry and Catalysis, Institute for Sustainable and Circular Chemistry, Faculty of Science, Utrecht University, Universiteitsweg 99 3584 CG Utrecht The Netherlands m.moret@uu.nl; b Structural Biochemistry, Bijvoet Centre for Biomolecular Research, Utrecht University 3584 CG Utrecht The Netherlands

## Abstract

A formal carbene-transfer reaction from an isolated nickelacyclobutane to an isocyanide to form a ketenimine is reported. DFT calculations support a stepwise 1,1-insertion/fragmentation pathway without a carbene intermediate. This unusual reactivity suggests a potential new role as “carbene reservoir” for nickelacyclobutanes, which are typically seen as intermediates in catalytic cyclopropanation.

## Abstract 2

A carbene-transfer reaction from an isolated nickelacyclobutane to an isocyanide to form a ketenimine is reported.

## Graphical abstract

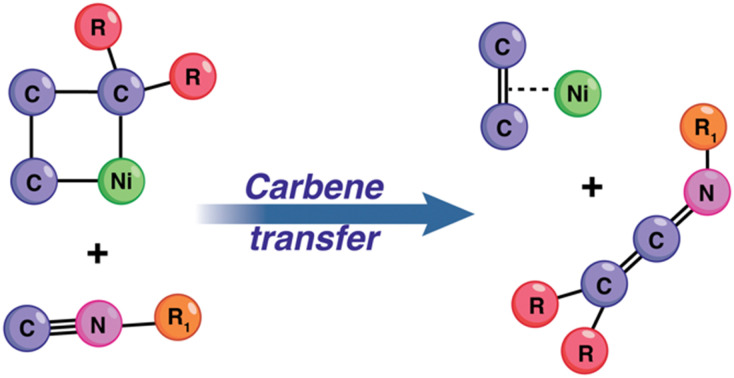

Metallacyclobutanes are often invoked as intermediates in catalytic cyclopropanation and olefin metathesis.^1–8^ Generally formed by [2+2] cycloaddition of a metal-carbene and an olefin, they are versatile intermediates that can undergo reductive elimination yielding cyclopropanes, [2+2] cycloreversion yielding a metal carbene and an olefin, and insertion of a neutral fragment yielding a metallacyclopentane (Fig. 1).^1–8^ As part of environmentally-motivated research efforts on base metal catalysis,^9,10^ Ni-catalyzed cyclopropanation has seen promising developments, where nickelacyclobutanes are proposed as key intermediates.^5–8,11–23^ To further our understanding of the reactivity of these species, we recently described the preparation of a pentacoordinated nickelacyclobutane embedded in a diphosphine pincer ligand.^7^ We found that exogenous ligands could selectively induce cyclopropanation (with the π-acceptor CO) or olefin-metathesis-like opening (with the σ-donor MeCN), in contrast with previously reported square planar nickelacyclobutanes.^8,16,22,24^

**Fig. 1:**
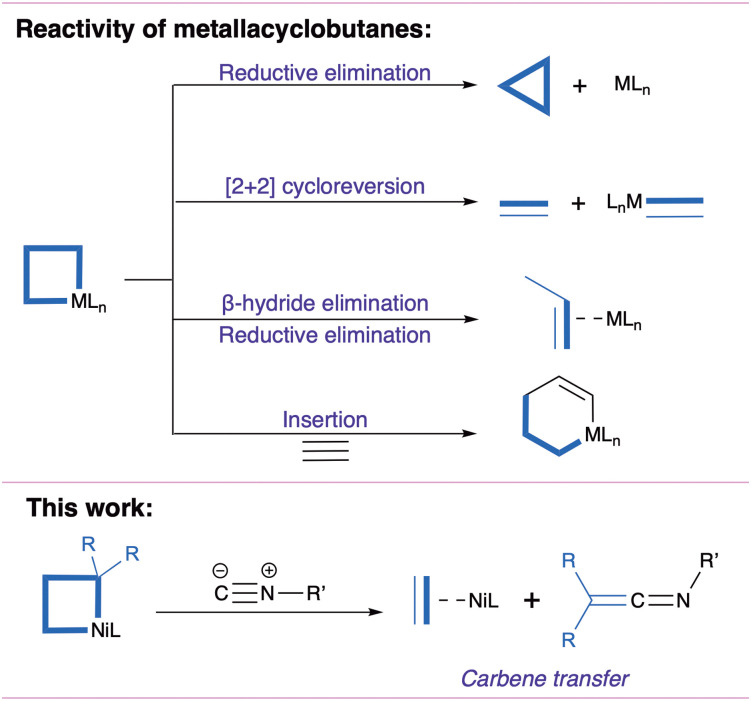
Reactivity of metallacyclobutanes and present work.

Here we report on an unexpected reactive pathway induced by coordination of *t*-butyl isocyanide (CN^*t*^Bu, R–NC): a formal carbene transfer generating a ketenimine (Fig. 1) and an olefin complex. While this process could be thought of as the result of [2+2] cycloreversion followed by coupling of the resulting nickel carbene and the isocyanide,^25–30^ DFT calculations support a distinct mechanism involving a nickelacyclopentane intermediate formed by 1,1-insertion of CN^*t*^Bu in a Ni–C bond. It suggests that these intermediates could act as “carbene reservoirs” and undergo carbene transfer reactions without prior [2+2] cycloreversion.

Reaction of the pentacoordinate nickelacyclobutane 1 with two equiv of CN^*t*^Bu in C_6_D_6_ initially resulted in rapid coordination of CN^*t*^Bu in apical position to yield 1-CN^*t*^Bu (Scheme 1). This is evidenced by a downfield shift and sharpening of the ^1^H NMR signal corresponding to the CH_2_ group from *δ* 4.40 ppm in 1 to 4.78 ppm in 1-CN^*t*^Bu, and by a sharpening of the ^31^P{^1^H} NMR signal to a singlet at 27.6 ppm (ESI,† Section S3). Both observations are consistent with the displacement of the π-interacting tolyl group by an isocyanide molecule to form a symmetrical structure and parallel those made upon coordination of CO.^7^

**Scheme 1:**
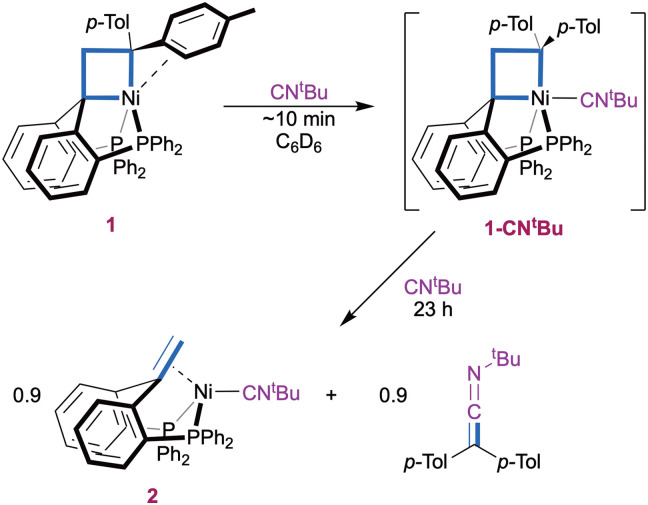
Carbene transfer with 2 eq. *t*-butylisocyanide.

A ^1^H NMR spectrum recorded after 2 h (ESI,† Section S2.2) showed the appearance of the olefin complex (^Ph^bppe^H,H^)Ni(CN^*t*^Bu) 2 and ketenimine *t*-BuN=C=C(*p*-Tol)_2_, as a result of the transfer of the C(*p*-Tol)_2_ carbene fragment to an isocyanide molecule. The reaction was complete after 23 h. Additionally, a small amount of 1,1-di(*p*-tolyl)ethylene was observed (∼10%) suggesting [2+2] cycloreversion as a minor pathway. Complex 2 was identified by a ^31^P{^1^H} NMR singlet at *δ*(C_6_D_6_) 25.7 ppm and a characteristic ^1^H NMR signal at *δ* 3.65 ppm (*t*, *J*_H,P_ = 2.2 Hz, 2H), that both match those of a sample of 2 independently synthesized from ^Ph^bppe^H,H^, Ni(cod)_2_ and CN^*t*^Bu (ESI,† Section 1.3 and 3). An ATR-FTIR spectrum of the reaction mixture (ESI,† Section S2.2) confirms the presence of complex 2 and displays the characteristic N=C=C stretching peak of *t*-BuN=C=C(*p*-Tol)_2_ as a strong signal at 2005 cm^−1^.^31,32^ The identity of the organic product is bolstered by the presence of ketenimine peak at 183.5 ppm in the APT ^13^C{^1^H} spectrum of the reaction mixture (ESI,† Section S2.2).^27,31,33^ Using a large excess of CN^*t*^Bu (55 equivalents) did not result in any substantial changes in the reactivity (ESI,† Section S2.3). No further reaction was observed when the isolated product 2 was exposed to bis(4-tolyl)diazomethane, indicating that the CN^*t*^Bu ligand in 2 binds too strongly for catalytic turnover to be accessible with this system.

More insights into the reaction mechanism were provided by an experiment with a lower amount (1.5 equiv.) of CN^*t*^Bu (Scheme 2). A slight excess was found necessary to ensure full initial conversion to 1-CN^*t*^Bu. Monitoring the reaction over time by ^31^P{^1^H} NMR again showed gradual conversion of 1-CN^*t*^Bu (*δ*(C_6_D_6_) 27.6 ppm) to compound 2 (*δ*(C_6_D_6_) 25.7 ppm) at early stages. However, a new P-containing species (3) appeared as a slightly broad singlet at *δ*(C_6_D_6_) 18.5 ppm after 1 h and was present in a 1 : 1.1 ratio with 2 after 18 h when all 1-CN^*t*^Bu was consumed (ESI,† Section S2.1). At this time, the concentration of ketenimine was approximately equal to the sum of those of complexes 2 and 3 according ^1^H NMR integration (Fig. S3, ESI†). As before, a small amount of 1,1-di(*p*-tolyl)ethylene (∼10%) was observed. Complex 3 is proposed to be a (^Ph^bppe^H,H^)Ni(L) type Ni(0) complex (*e.g.* L = C_6_D_6_) on the basis of its NMR characteristics. Namely, a broad ^1^H NMR singlet at *δ*(C_6_D_6_) 3.79 ppm is consistent with a Ni-bound olefinic CH_2_ group. ^1^H-^31^P HMBC confirmed that the signal at *δ*(C_6_D_6_) 3.79 ppm is related to the ^31^P{^1^H} NMR peak at 18.5 ppm. The identity of complex 3 was further confirmed by quenching the reaction mixture with 1.5 equivalents of PPh_3_, which resulted in full conversion of complex 3 to (^Ph^bppe^H,H^)Ni(PPh_3_) (4) while complex 2 remained unaffected (Fig. S6 and S7, ESI†). Complex 4 was identified by comparison with a sample independently synthesized from the ^Ph^bppe^H,H^ ligand, Ni(cod)_2_, and PPh_3_ (ESI,† Section 1.3 and 3), and it is molecular structure was confirmed by X-ray crystal structure determination in addition to its spectroscopic identification (ESI,† Section 4). These results indicate that the second equivalent of CN^*t*^Bu in Scheme 1 is not required for the carbene transfer step itself, but simplifies the final reaction mixture by capturing the formed Ni(0) fragment.

**Scheme 2:**
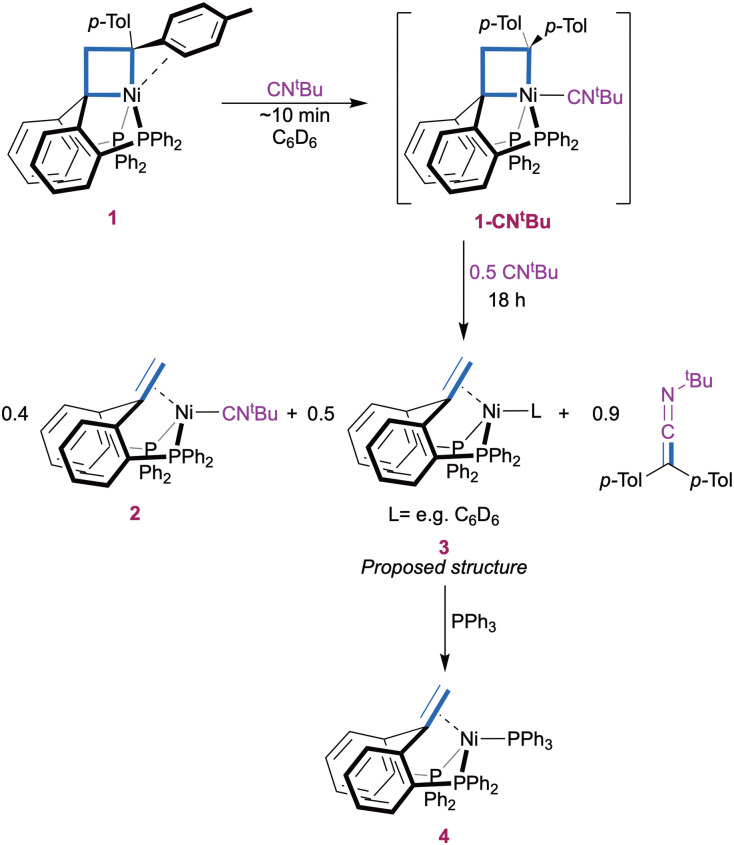
Carbene transfer with 1.5 eq. *t*-butylisocyanide.

Ketenimines are versatile compounds in organic synthesis,^29,31,34–36^ which can be synthesized, amongst other, by (catalytic) coupling of a metal carbene and an isocyanide molecule.^25–30^ This could suggest a mechanism in which reversible [2+2] cycloreversion of the pentacoordinated nickelacyclobutane generates a carbene fragment that is intercepted by the isocyanide reagent. A similar [2+2] cycloreversion has been proposed by Miyashita to explain the reaction of the tetracoordinated nickelacyclobutane (PPh_3_)_2_Ni(2,2-dimethylpropa-1,3-diyl) with CO or cyclohexene to generate ketene (O=C=CH_2_) or the cyclopropanation product bicyclo[4.1.0]heptane, respectively.^21,22,37^ In a somewhat related report, Neely and coworkers described an iron azametallacyclobutene with a significant iron carbene resonance, which reacts with isocyanide and CO to form ketenimines and ketenes.^38^ On the other hand, isocyanides have also been known to undergo migratory insertion with metallacyclobutanes to yield metallacyclopentanes for several metals.^8,39–44^ In the next section, we assess the feasibility of these different processes using DFT calculations^45^ performed using a slightly truncated model with Ph groups instead of *p*-Tol.

We found the process with the lowest overall barrier to start with 1,1-insertion of the isocyanide ligand in a M–C bond of the metallacyclobutane to expand the ring (Fig. 2a). Starting from complex 1-CN^*t*^Bu, insertion to yield nickelacyclopentane 5 (with the nitrogen lone pair opposite to nickel) is energetically accessible (Δ*G*^‡^ = 25.6 kcal mol^−1^), followed by reductive fragmentation (Δ*G*^‡^ = 25.7 kcal mol^−1^) forming complex 6. Change of coordination of the ketenimine from *η*^2^(C,C) to *η*^1^(N) yields complex 6 a more stable isomer (−1.9 kcal mol^−1^, ESI† Section S5.2). If an excess of isocyanide is available, ligand exchange to form complex 2 yields an overall free energy release of −19.8 kcal mol^−1^. Alternative routes starting with 1,1-insertion were found to be less favorable (ESI,† Section S5.3). The overall barrier of 25.7 kcal mol^−1^ is at the upper bound for a slow process at room temperature and significantly lower than all other considered pathways.^46^

**Fig. 2:**
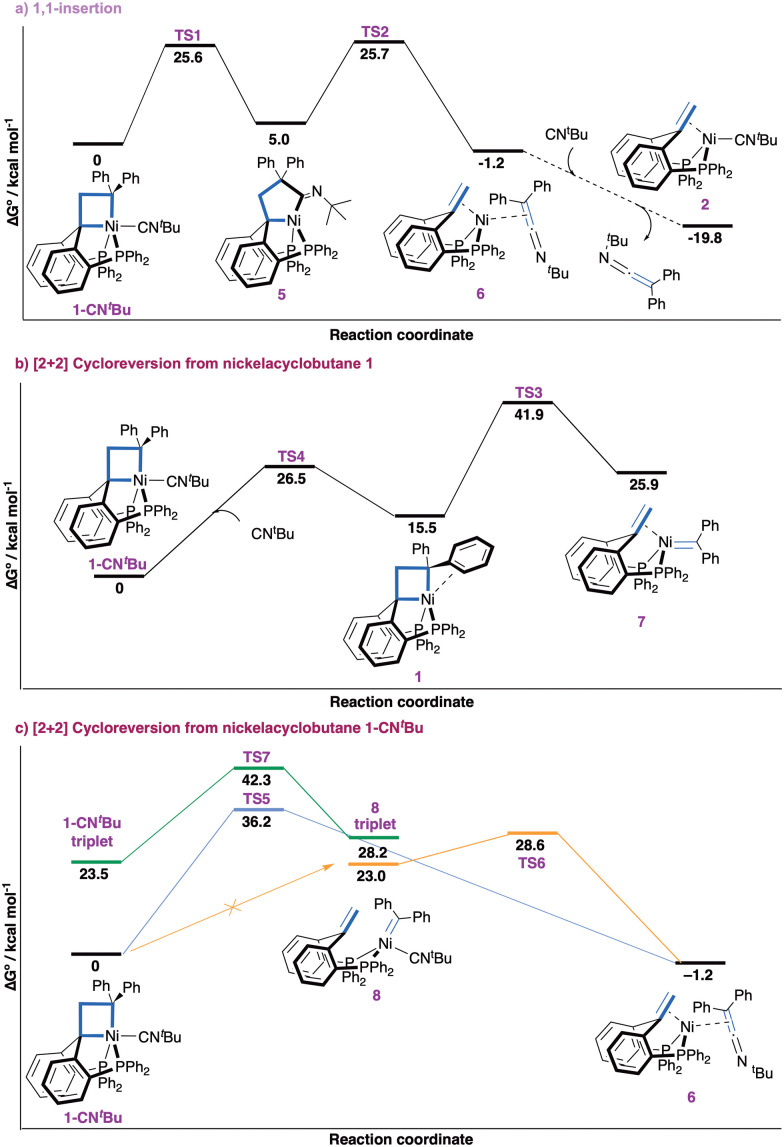
Δ*G*° energy profiles for the reactivity of 1-CN^*t*^Bu*via* 1,1-insertion (a) or [2+2] cycloreversion (b and c) mechanisms. Dashed lines connect intermediates between which no transition state was optimized.

Pathways involving a [2+2] cycloreversion process yielding a nickel carbene intermediate were calculated to be energetically inaccessible (Fig. 2b). First, the formation of the putative carbene/olefin species 7 from the observed adduct 1-CN^*t*^Bu after ligand dissociation is kinetically inaccessible. Initial decoordination of CN^*t*^Bu to form nickelacyclobutane 1 is endergonic by 15.5 kcal mol^−1^ and hampered by a barrier of 26.5 kcal mol^−1^. [2+2] Cycloreversion to 7 comes with an additional endergonicity of 10.4 kcal mol^−1^ and a prohibitively high overall barrier (Δ*G*^‡^ = 41.9 kcal mol^−1^). An alternative process starting with decoordination of one phosphine arm of nickelacyclobutane 1 was discarded due to the high energy of this ligand dissociation (27.6 kcal mol^−1^, ESI† Section S5.3). Second, we investigated whether the carbene fragment could be directly transferred to the CN^*t*^Bu ligand in 1-CN^*t*^Bu (Fig. 2c). A transition state for concerted carbene transfer was located yielding complex 6 (−1.2 kcal mol^−1^), but the associated barrier is prohibitively high (Δ*G*^‡^ = 36.2 kcal mol^−1^). Third, a nickel carbene complex 8 bearing an isocyanide ligand was found to be relatively high in energy (23.0 kcal mol^−1^). Attempts to locate a transition state for the [2+2] cycloreversion yielding 8 from 1-CN^*t*^Bu were unsuccessful. A potential energy surface (PES) scan suggests there is actually no transition state connecting complex 8 to 1-CN^*t*^Bu (Fig. S35, ESI†). Rather, the ketenimine complex 6 appears to be an intermediate in the hypothetical transformation of 1-CN^*t*^Bu into 8. This suggests complex 8 is not an intermediate of the process. Additionally, we disfavor complex 8 as a plausible intermediate due to the high free energy (Δ*G*^‡^ = 28.6 kcal mol^−1^) of the transition state for the formation of ketenimine complex 6 from 8. Fourth, the possibility of two-state reactivity involving the triplet state was also considered,^47^ but the [2+2] cycloreversion process in triplet state was associated with a prohibitively high barrier (Δ*G*^‡^ = 42.3 kcal mol^−1^). Finally, the direct carbene transfer and [2+2] cycloreversion process starting from a tetracoordinated nickelacyclobutane (1-CN^*t*^Bu-noP) resulting from decoordination of one phosphine arm was computed (ESI,† Section 5.4). Both processes were found unfeasible with overall barriers of Δ*G*^‡^ = 52.1 kcal mol^−1^ and Δ*G*^‡^ = 48.3 kcal mol^−1^, respectively. Hence, no energetically accessible pathway for direct carbene transfer with or without a nickel carbene intermediate was identified, further supporting the sequential 1,1-insertion/reductive fragmentation as operative mechanism for the observed formal carbene transfer reaction.

The contrasting reactivity of 1-CN^*t*^Bu (carbene transfer) and 1-CO (cyclopropane formation)^7^ is surprising in view of the isoelectronic character of CO and isocyanides. To obtain additional insights, the different decomposition pathways were investigated computationally for both compounds (ESI,† Sections S5.5 and S5.6). For 1-CN^*t*^Bu the transition state for cyclopropane formation by reductive elimination was found to be prohibitively high in energy (Δ*G*^‡^ = 33.9 kcal mol^−1^) in good agreement with experiment. The calculated barrier of 22.5 kcal mol^−1^ for [2+2] cycloreversion yielding (PC_carbene_P)Ni(CN^*t*^Bu) is *ca.* 3 kcal mol^−1^ lower than that for isocyanide insertion. The disparity is at odds with experimental observations but within the typical error range of DFT calculations. Additionally, the experimental observation of a small amount of 1,1-di(*p*-tolyl)ethene alongside the carbene transfer process is consistent with a small difference between the barriers for [2+2] cycloreversion and insertion. For 1-CO, cyclopropane formation is the favoured reaction pathway with an overall barrier of 23.8 kcal mol^−1^ in good agreement with experiment. The [2+2] cycloreversion process is higher in energy by 4 kcal mol^−1^ and insertion pathway is higher by 6.1 kcal mol^−1^. These differences highlight the high sensitivity of the pentacoordinated nickelacyclobutane 1 towards the electronic nature of the exogeneous ligand in apical position, the stronger π-accepting character of CO markedly favouring reductive elimination of a cyclopropane unit.

In summary, we disclose an unusual carbene transfer reaction from a pentacoordinated nickelacyclobutane to a molecule of CN^*t*^Bu yielding a ketenimine. DFT calculations support a mechanistic pathway that does not involve a nickel carbene intermediate but instead a nickelacyclopentane resulting from 1,1-insertion of CN^*t*^Bu into a Ni–C bond. These results further highlight the importance of the coordination environment of nickelacyclobutane intermediates for selective reactions. The possibility to access carbene-like reactivity without an actual carbene intermediate (*e.g.* generated by [2+2] cycloreversion) suggests a possible use of metallacyclobutanes as “carbene reservoirs” to tame unstable metal carbene fragments.

This project has received funding from the European Research Council (ERC) under the European Union's Horizon 2020 research and innovation program (grant agreement No 715060). The X-ray diffractometer has been financed by the Netherlands Organization for Scientific Research (NWO). This work made use of the Dutch national e-infrastructure with the support of the SURF Cooperative using grant no. EINF-3520. The authors thank Storm van der Voort, for his assistance in the synthesis of complex 4; and Dr Andrea Darú, for his assistance with DFT calculations.

## Data availability

The supplementary data of this article have been included in the ESI† contains synthesis and characterization of new compounds, additional experiments and computational details. CCDC number 2365514† contains supplementary crystallographic data that can be obtained at the Cambridge Crystallographic Data Centre.

## Conflicts of interest

There are no conflicts to declare.

## Supplementary Material

CC-060-D4CC04273E-s001

CC-060-D4CC04273E-s002

CC-060-D4CC04273E-s003
