# Supplementary material for: Carbene transfer reactivity from a nickelacyclobutane
Source: Chem Commun (Camb). 2024 Sep 30;60(85):12397–400. doi: 10.1039/d4cc04273e (PMC11456996; doi:10.1039/d4cc04273e)
Supplement: CC-060-D4CC04273E-s002 [file CC-060-D4CC04273E-s002.pdf]

Supplementary Information (SI) for ChemComm.
This journal is © The Royal Society of Chemistry 2024

Supporting information

María L. G. Sansores-Paredes[1], Martin Lutz[2] and Marc-Etienne Moret[1]*

[1]Organic Chemistry & Catalysis, Institute for Sustainable and Circular Chemistry, Utrecht University, 3584 CG, Utrecht, The Netherlands.

[2]Structural Biochemistry, Bijvoet Centre for Biomolecular Research, Utrecht University, 3584 CG, Utrecht, The Netherlands.

Email corresponding author: m.moret@uu.nl

TABLE OF CONTENTS

1. Experimental section **S1**

1.1 General information S1

1.2 Physical methods S1

1.3 Synthesis and characterization S1

2. Additional experiments **S4**

2.1 Reaction of Nickelacyclobutane (1) and 1.5 equivalents of *t*-butylisocyanide S4

2.2 Reaction of Nickelacyclobutane (1) and 2 equivalents of *t*-butylisocyanide S9

2.3 Reaction of Nickelacyclobutane (1) and 55 equivalents of *t*-butylisocyanide S13

3. Spectra of new compounds **S16**

4. X-ray crystal structure determination **S27**

5. DFT studies **S28**

5.1 General information S28

5.2 Isomers of complex 6 .... S28

5.2.1 1,1-insertion route towards other nickelacyclopentane isomer S29

5.2.2 1,1-insertion route involving two molecules of *t*-butylisocyanide S29

5.2.3 1,1-insertion route towards in triplet state S30

5.3 Additional information of [2+2] Cycloreversion route S31

5.4 Additional 1-CN^{t}Bu routes S32

5.5 Comparison with 1-CO S33

5.6 Table of energies S33

6. Literature references **S35**

1. Experimental section

1.1 General information

All reactants were purchased from commercial sources and used as received without further purification. Additionally, $Ni(cod)_2$, PPh_3 and *t*-butyl isocyanide were stored in the glovebox. All the reactions were performed under N_2(g) atmosphere in a glovebox. Deuterated solvents were purchased from Cambridge Isotope Laboratory Incorporation (Cambridge, USA), degassed by freeze pump procedure, and stored over molecular sieves before use. Common solvents were dried using a MBRAUN MB SPS-80 purification system. Nickelacyclobutane (**1**) and ligand ^{Ph}bppe^{H,H} were synthetized according to literature procedures.[1]

1.2 Physical methods

^{1}H, ^{13}C and ^{31}P NMR spectra (400, 101, and 161 MHz respectively) were recorded on an Agilent MR400 or a Varian AS400 spectrometer at 25 ºC. ^{1}H and ^{13}C NMR chemical shifts relative to tetramethylsilane are referenced to the residual solvent resonance. ^{31}P NMR chemical shifts were referenced to 85% aqueous H_3PO_4 solution externally. Infrared spectra were recorded using a Perking Elmer Spectrum One FT-IR spectrometer under N_2 flow. The bulky purity of the reported compounds is supported by clean NMR spectra.

1.3 Synthesis and characterization

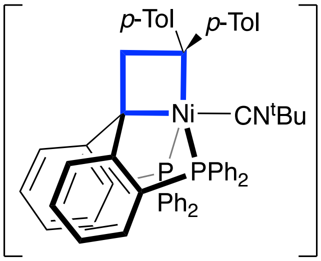

1-CN^{t}Bu: Inside the drybox, nickelacyclobutane **1** (8 mg, 0.01 mmol) was dissolved in C_6D_6 (0.6 mL). Then, 45 µL of a 0.3 M solution of *t*-butylisocyanide in C_6D_6 (0.015 mmol) were added, causing a lightening from a dark red to a clearer red solution. The solution was transferred to a J. Young NMR tube. After 10 min, NMR data were recorded. ^{1}H NMR (400 MHz, C_6D_6, 25 ºC): δ(ppm) 7.96 (d, *J*= 8.6 Hz, 3H, Ar-*H*), 7.28 (d, *J*= 8.3 Hz, 4H, Ar-*H*), 6.97 (m, 13H, Ar-*H*), 6.85 (t, *J*= 7.4 Hz, 3H, Ar-*H*), 6.81 (t, *J*= 7.3 Hz, 3H, Ar-*H*), 6.75 (d, *J*= 8.3 Hz, 4H, Ar-*H*), 6.67 (t, *J*= 7.8 Hz, 4H, Ar-*H*), 4.78 (s, 2H, C*H_2*), 2.15 (s, 6H, C*H_3*), 0.94 (s, 9H, C(C*H_3*)$_3$). Some aromatic signals overlap with the residual peak of the deuterated solvent

^{31}P{^{1}H} NMR (162 MHz, C_6D_6, 25 ºC): δ(ppm) 27.6 (s, 2P).

The complex is thermally unstable with a half-life of approximately 1 hour in C_6D_6 (see Figure S2), it did not allow to obtain ^{13}C NMR, IR, and elemental analysis data.

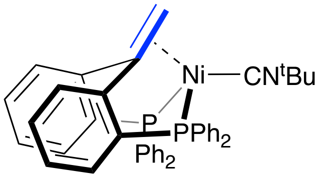

(^{Ph}bppe^{H,H})Ni(CNtBu) (**2**): Ligand ^{Ph}bppe^{H,H} (50 mg, 0.09 mmol) was placed in a vial and dissolved in toluene (5 mL). A solution of Ni(cod)$_2$ (25 mg, 0.09 mmol) in toluene (3 mL) was added in one portion. The reaction mixture was stirred for 5 min and a solution of *t*-butylisocyanide (7.5 mg, 0.09 mmol) in toluene (0.5 mL) was added. The mixture was then stirred at room temperature for 4 h. After this, the volume of solvent was reduced *in vacuo* to a quarter of the original volume and the product was precipitated using hexane (4 mL). The solid was filtered, washed (3 x 1 mL) with cold hexane (cooled down in the glovebox freezer at –35 °C), and dried to yield 56 mg of a yellow powder with 91% yield.

^{1}H NMR (400 MHz, C_6D_6, 25 °C): δ(ppm) 8.03–7.95 (m, 4H, Ar-*H*), 7.90 (dt, *J* = 7.8, 1.6 Hz, 2H, Ar-*H*), 7.22–7.17 (m, 5H, Ar-*H*), 7.14-7.12 (m, 1H, Ar-*H*), 7.05 (t, *J* = 7.5 Hz, 2H, Ar-*H*), 7.01–6.95 (m, 4H, Ar-*H*), 6.93–6.84 (m, 4H, Ar-*H*), 6.83–6.77 (m, 4H, Ar-*H*), 3.65 (t, $J_{H,P}$ = 2.2 Hz, 2H, CH_2), 1.05 (s, 9H, CH_3). Some aromatic signals overlap with the residual peak of the deuterated solvent.

^{31}P{^{1}H} NMR (162 MHz, C_6D_6, 25 °C): δ(ppm) 25.7 (s, 2P).

^{13}C{^{1}H} NMR (101 MHz, C_6D_6, 25 °C): δ(ppm) 166.0 (s, Ar), 158.3–155.0 (m, Ar or *C*N), 142.5–141.8 (m, Ar or *C*N), 140.5–140.0 (m, Ar), 139.8–139.1 (m, Ar), 133.9 (t, *J* = 8.0 Hz, Ar), 133.2 (s, Ar), 132.5–131.9 (m, Ar), 128.6 (s, Ar), 128.3 (s, Ar), 127.1 (s, Ar), 126.3 (d, *J* = 2.0 Hz, Ar), 97.5 (t, *J* = 5.0 Hz, CH_2=*C*), 59.9 (t, *J*=7.2 Hz, *C*H_2), 55.1 (s, *C*(CH_3)$_3$), 30.8 (s, *C*H_3). Some aromatic signals overlap with the residual peak of the deuterated solvent.

IR (cm^{-1}): 3050, 2977, 2928, 2858, 2094 (sh), 2059 (ν CN), 1583, 1478, 1460, 1433, 1366, 1212, 1122, 1090, 1067, 909, 778, 742, 695, 604, 515, 504.

The high sensitivity of this compound did not allow to obtain elemental analysis data.

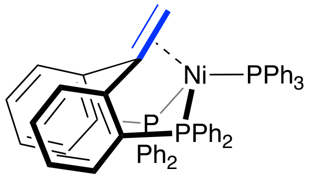

(^{Ph}bppe^{H,H})Ni(PPh$_3$) (**4**): Ligand ^{Ph}bppe^{H,H} (20 mg, 0.036 mmol) was placed in a vial and dissolved in 2 mL of toluene. A solution of Ni(cod)$_2$ (10 mg, 0.036 mmol) in toluene (1 mL) was added in one portion. The reaction was stirred for 5 min and PPh_3 (9.5 mg, 0.0365 mmol) was added, after

which the mixture was stirred at room temperature for 4 h. Afterwards, the solvent was evaporated, and the residue was washed four times with 0.5 mL of cold hexane (cooled down in the glovebox freezer at –35 °C). The product was dried and isolated as 24 mg of an orange powder with 77% yield. Crystals suitable for X-ray diffraction were obtained at room temperature by vapor diffusion of hexane into a concentrated toluene solution.

^{1}H NMR (400 MHz, C_6D_6, 25 °C): δ(ppm) 7.84 (dd, J = 7.8, 1.9 Hz, 2H, Ar-H), 7.42–7.25 (m, 9H, Ar-H), 7.14–7.01 (m, 5H, Ar-H), 6.96–6.88 (m, 4H, Ar-H), 6.88–6.81 (m, 7H, Ar-H), 6.78 (t, J = 7.4 Hz, 3H, Ar-H), 6.70 (t, J = 7.5 Hz, 3H, Ar-H), 3.52 (dt, $J_{H,P}$ = 6.8, 2.4 Hz, 2H, CH_2). Some aromatic signals overlap with the residual peak of the deuterated solvent.

^{31}P NMR (162 MHz, C_6D_6, 25 °C): δ(ppm) 46.1 (t, $J_{P,P}$=22.3, 1P, PPh$_3$), 24.1 (d, $J_{P,P}$=22.3, 2P).

^{13}C NMR (101 MHz, C_6D_6): δ(ppm) 155.9 (d, J = 38.7 Hz, Ar), 143.1–142.2 (m, Ar), 139.6–139.2 (m, Ar), 139.1 (t, J = 5.7 Hz, Ar), 138.4 (t, J = 14.5 Hz, Ar), 134.4–133.4 (m, Ar), 132.8 (t, J = 6.5 Hz, Ar), 132.1 (s, Ar), 128.6 (s, Ar), 128.3 (s, Ar), 127.8–127.5 (m, Ar), 127.1 (s, Ar), 126.0 (s, Ar), 97.1 (dt, J = 13.3, 5.5 Hz, CH_2=C), 58.6 (m, CH$_2$=C). Some aromatic signals overlap with the residual peak of the deuterated solvent.

IR (cm^{-1}): 2457, 2923, 2853, 1477, 1458, 1432, 1260, 1086, 1026, 804, 738, 692, 514, 491, 426.

The high sensitivity of this compound did not allow to obtain elemental analysis data.

2. Additional experiments

2.1 Reaction of Nickelacyclobutane (1) and 1.5 equivalents of *t*-butylisocyanide

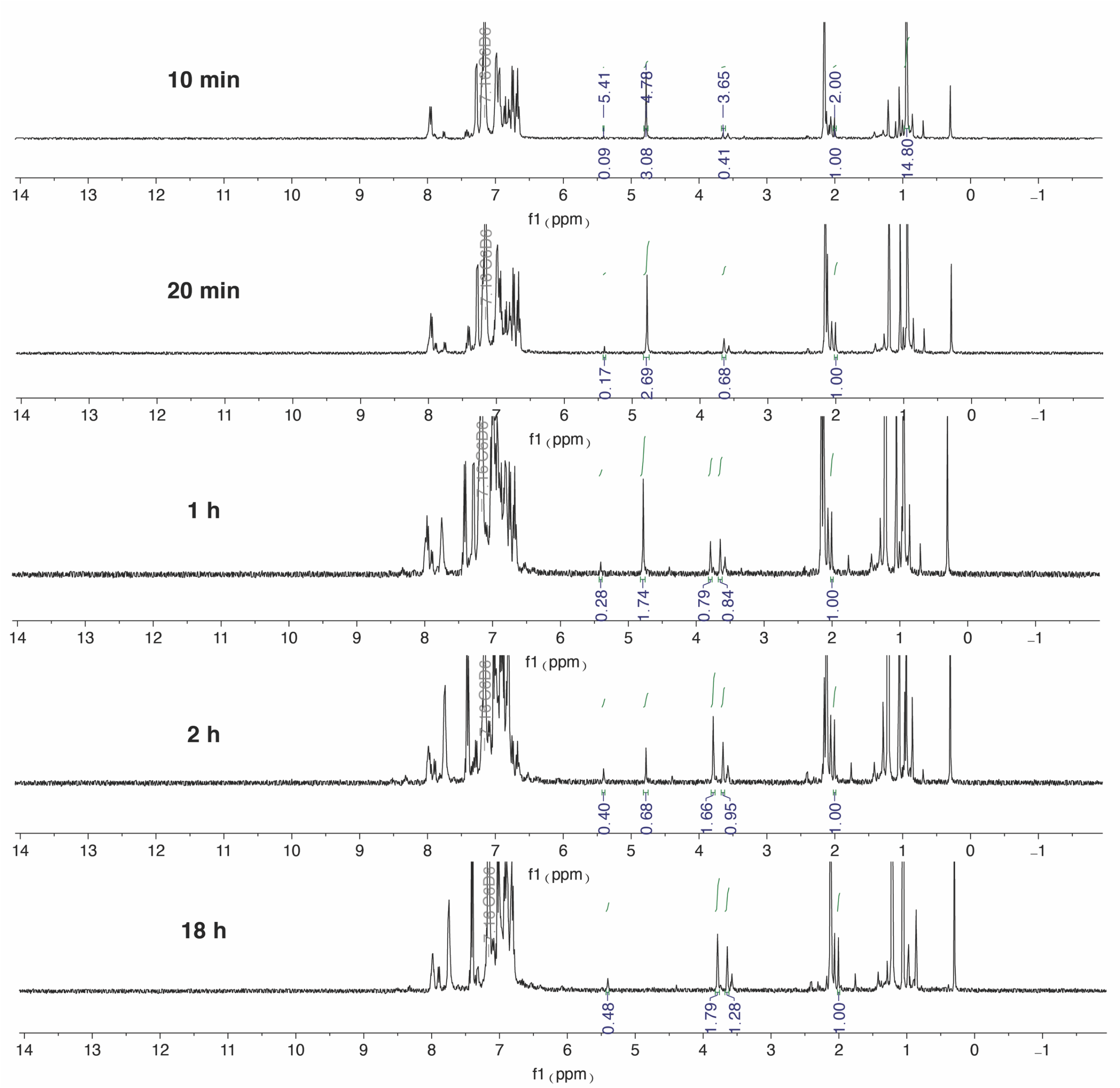

Figure S1. Full ^{1}H NMR spectra (range –2 - 14 ppm) of reaction between 1.5 equiv. of *t*-butylisocyanide and nickelacyclobutane **1** at different times. The peak at 2.00 ppm corresponds to the known impurity di-*p*-tolylketon-azin and was used as integration reference. The peak marked with an asterisk corresponds to 1,1-di(*p*-tolyl)ethene. The small peak at 3.5 ppm corresponds to residual THF.

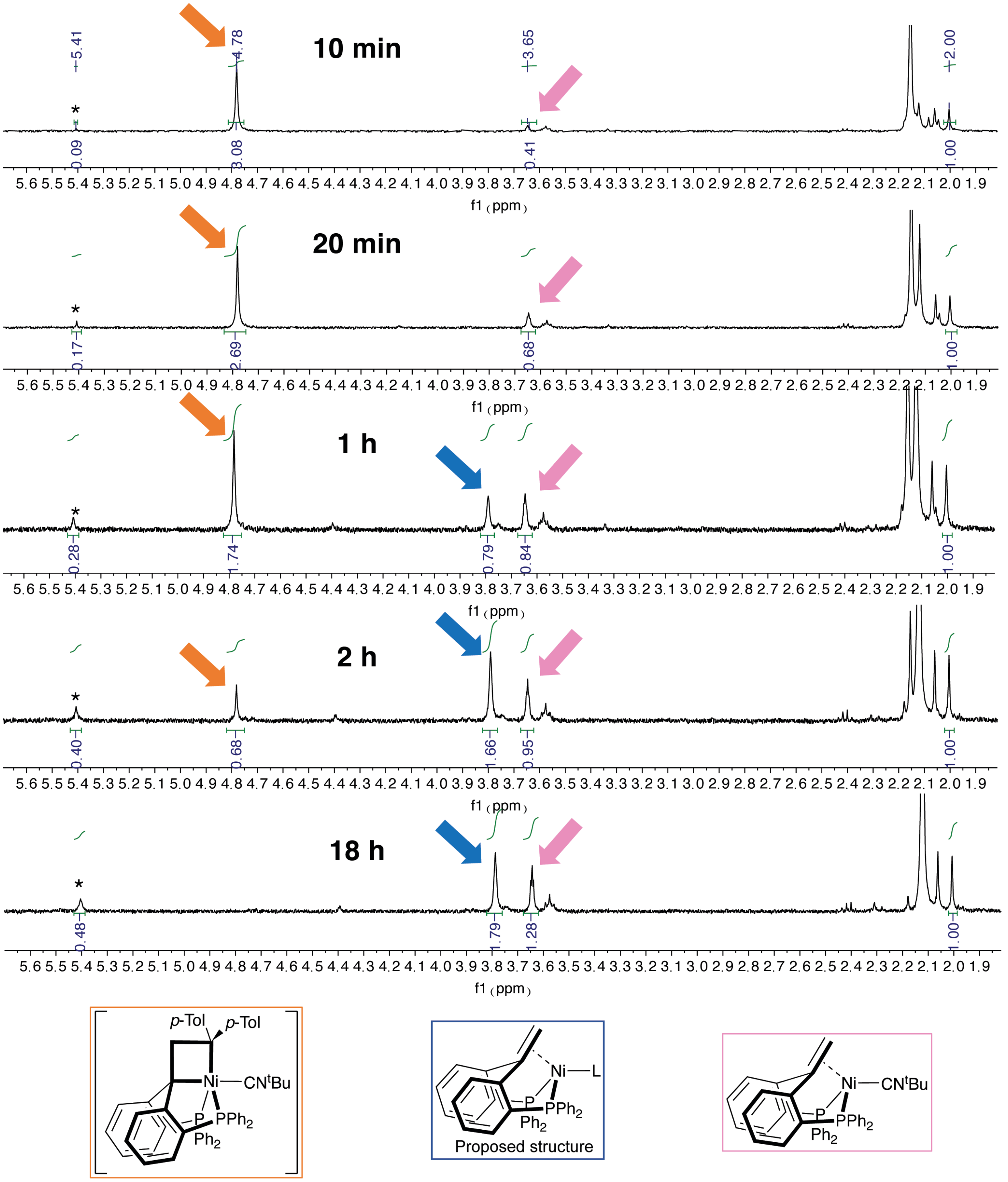

Figure S2. ^{1}H NMR spectra in C_6D_6 (range 1.8-5.7 ppm) of reaction between 1.5 equiv. of *t*-butylisocyanide and nickelacyclobutane **1** at different times. The peak at 2.00 ppm corresponds to the known impurity di-*p*-tolylketon-azin and was used as integration reference. The peak marked with an asterisk corresponds to 1,1-di(*p*-tolyl)ethene. The small peak at 3.5 ppm corresponds to residual THF.

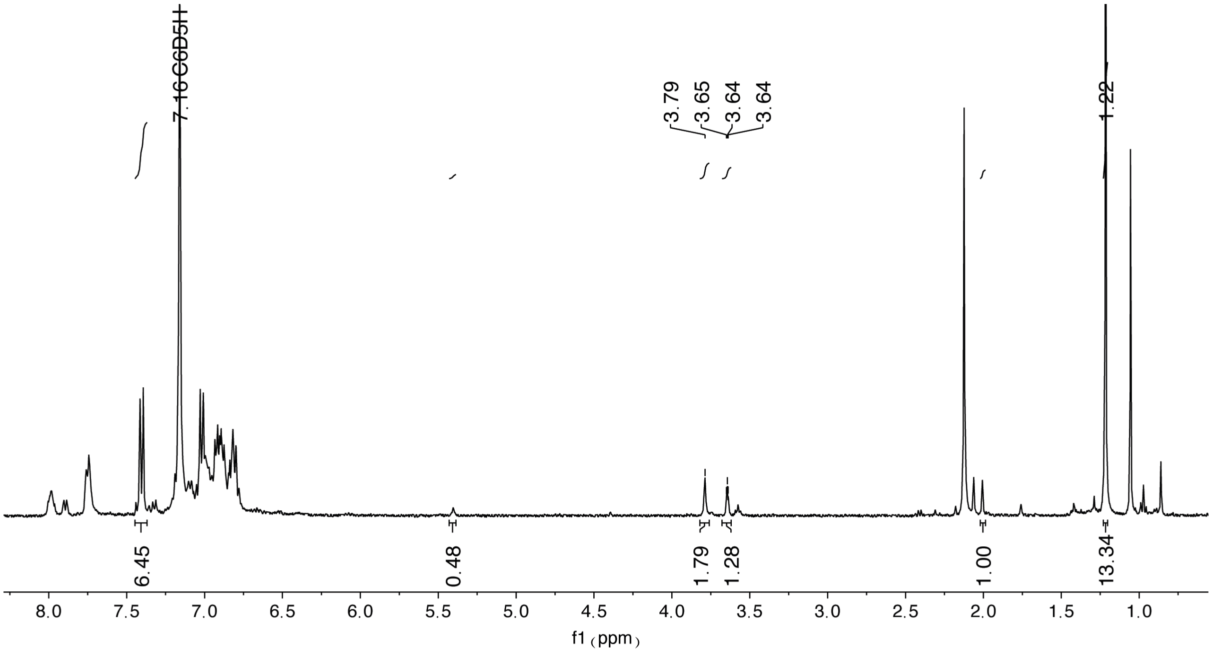

Figure S3. ^{1}H NMR spectrum in C_6D_6 of reaction between 1.5 equiv. of *t*-butylisocyanide and nickelacyclobutane **1** after 18 h.

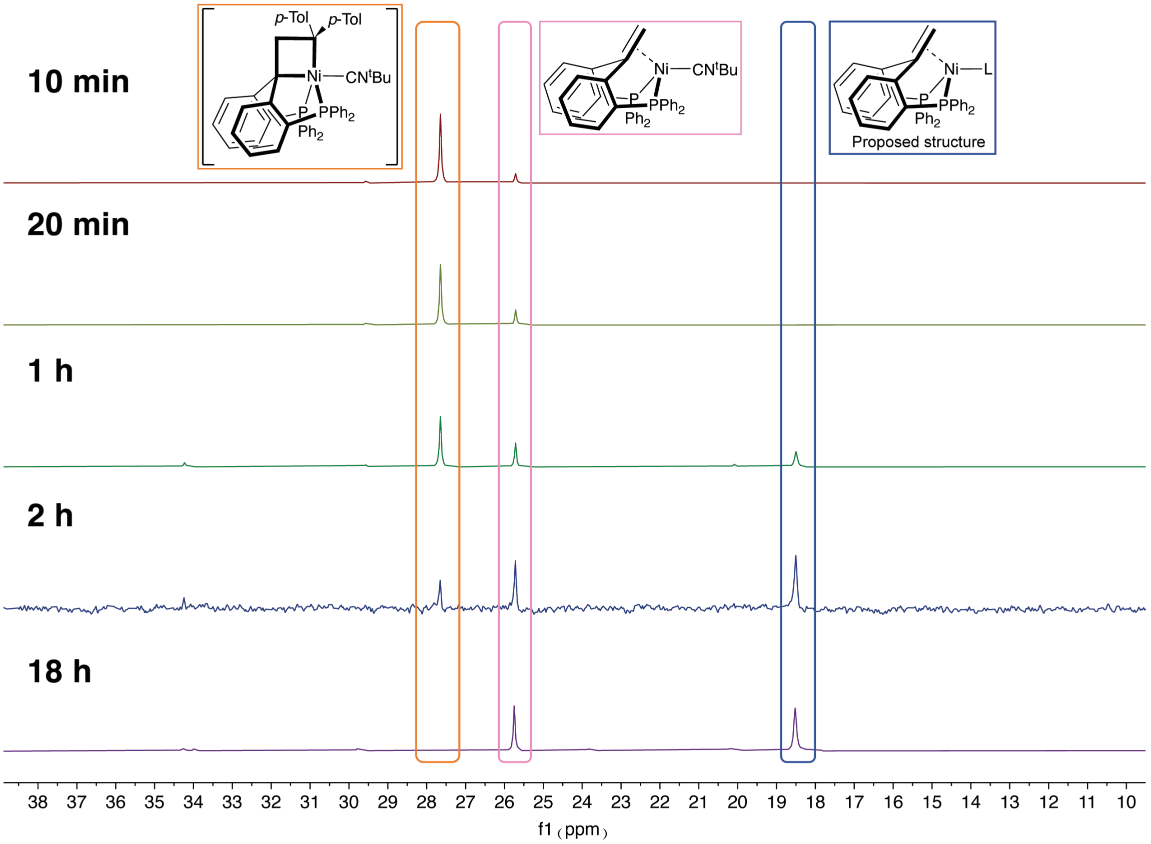

Figure S4. $^{31}P\{^{1}H\}$ NMR spectra in C_6D_6 of reaction between 1.5 equiv. of *t*-butylisocyanide and nickelacyclobutane **1** at different times.

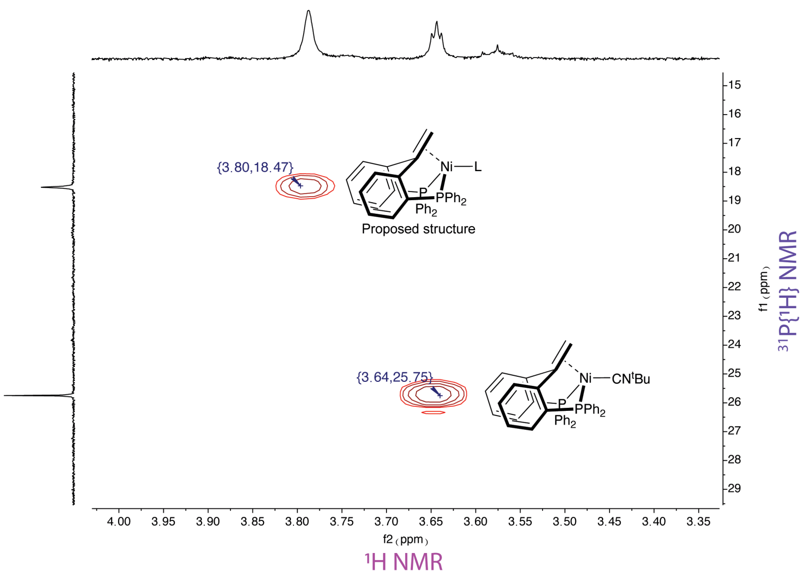

Figure S5. ^1H-^{31}P HMBC of reaction of nickelacyclobutane **1** with 1.5 equiv. of *t*-butylisocyanide after 18 h.

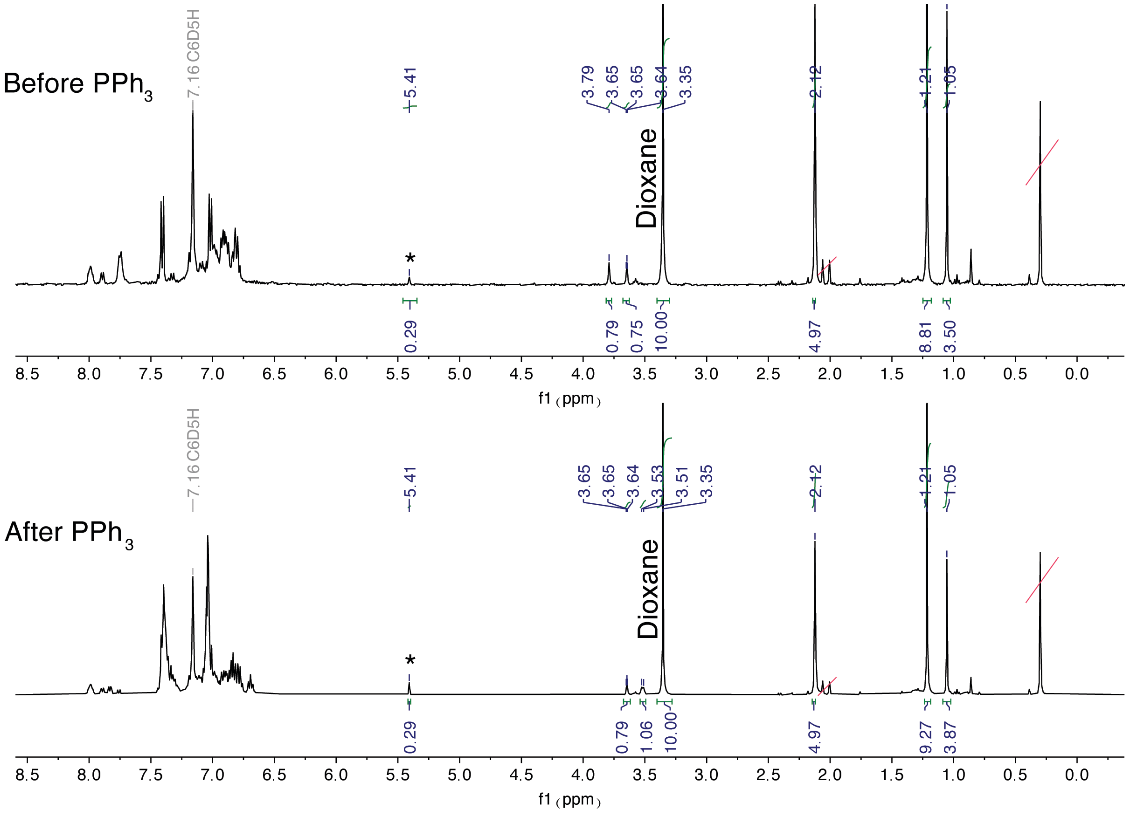

Figure S6. ^1H NMR in C_6D_6 of the addition of 1.5 equiv. of PPh_3 to a reaction mixture of nickelacyclobutane **1** with 1.5 equivalents of *t*-butylisocyanide after 18 h. Dioxane was added simultaneously as internal standard. Crossed peaks with red lines correspond to di-*p*-tolylketon-azin and silicon grease. The peak marked with an asterisk correspond to 1,1-di(*p*-tolyl)ethene. Characteristic ^1H NMR signal for the CH_2 moiety of **4**: 3.47 (dt, $J_{H,P}$= 6.8, 2.4 Hz).

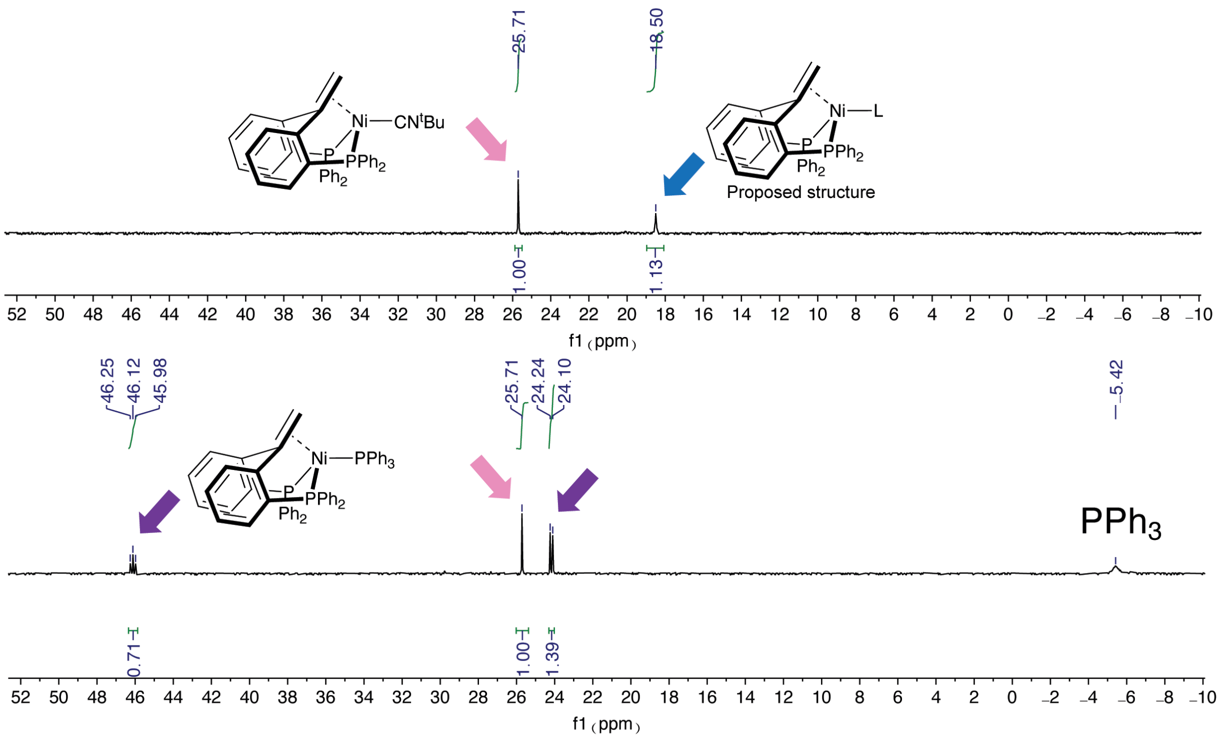

Figure S7. $^{31}P\{^{1}H\}$ NMR in C_6D_6 of the addition of 1.5 equiv. of PPh_3 to a reaction mixture of nickelacyclobutane **1** with 1.5 equivalents of *t*-butylisocyanide after 18 h. Characteristic $^{31}P\{1H\}$ NMR signals of **4**: 46.1 (t, $J_{P,P}$=22.3, 1P, PPh_3) and 24.1 (d, $J_{P,P}$=22.3, 2P).

2.2 Reaction of Nickelacyclobutane (1) and 2 equivalents of *t*-butylisocyanide

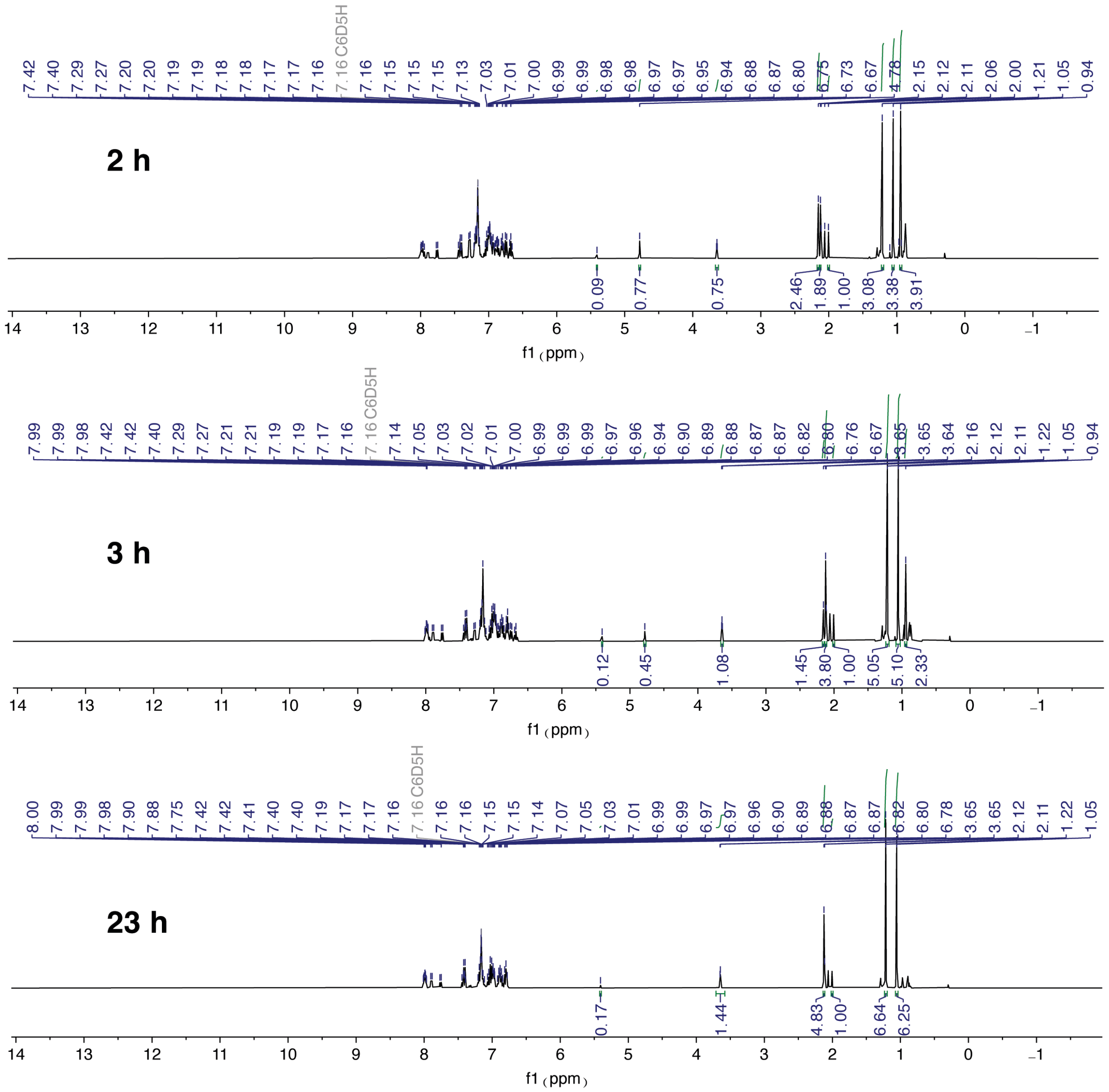

Figure S8. Full ^{1}H NMR spectra (range –2 - 14 ppm) of the reaction of nickelacyclobutane **1** and two equivalents of *t*-butylisocyanide in C_6D_6 at different times. The peak at 2.00 ppm corresponds to the known impurity di-*p*-tolylketon-azin and was used as integration reference.

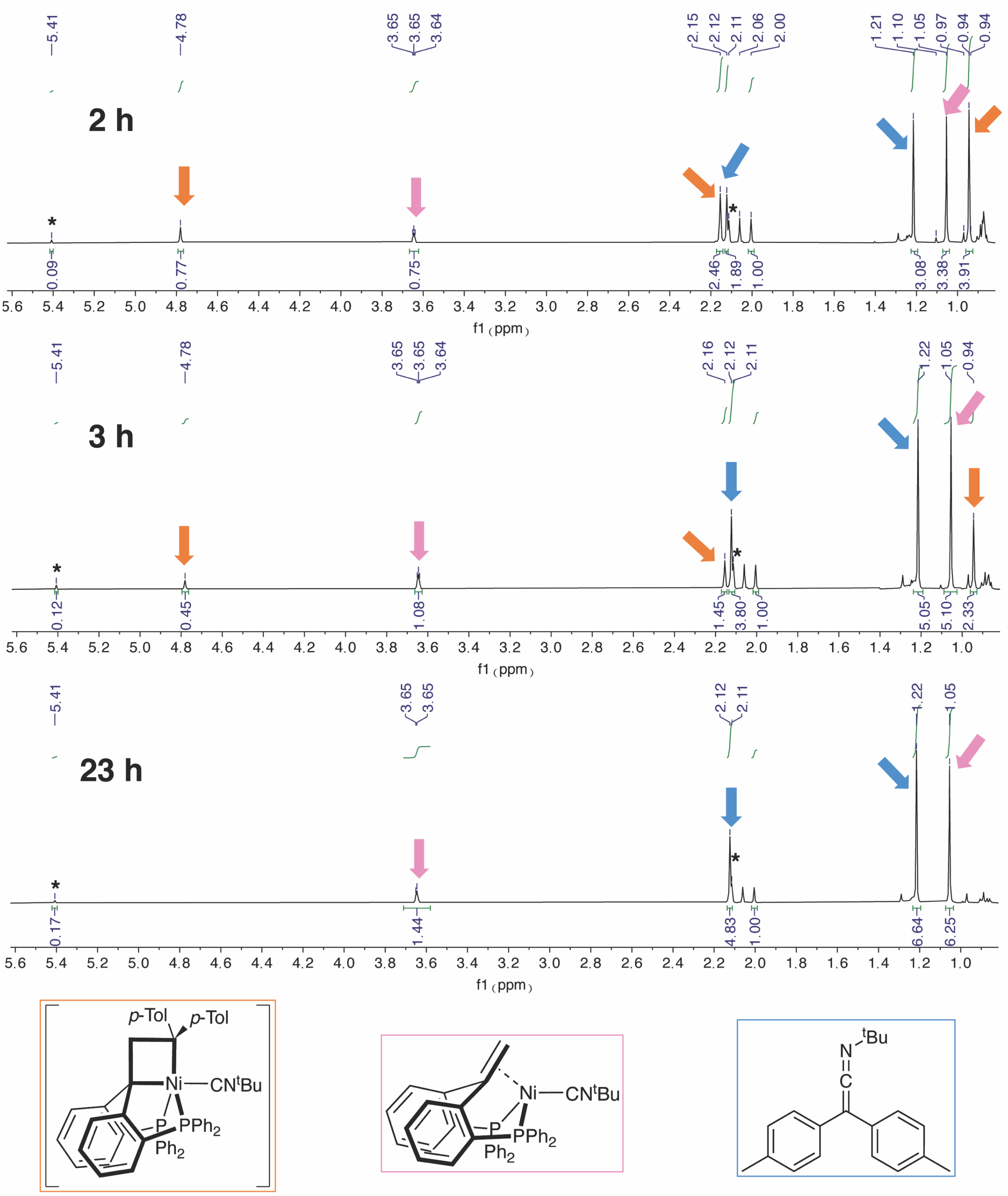

Figure S9. ^{1}H NMR spectra in C_6D_6 zoomed in the range 1.0-5.6 ppm of the reaction of nickelacyclobutane **1** and two equivalents of *t*-butylisocyanide at different times. The peaks marked with an asterisk correspond to 1,1-di(*p*-tolyl)ethene. The peak at 2.00 ppm corresponds to the known impurity di-*p*-tolylketon-azin originating from the synthesis of **1** and was used as integration reference.

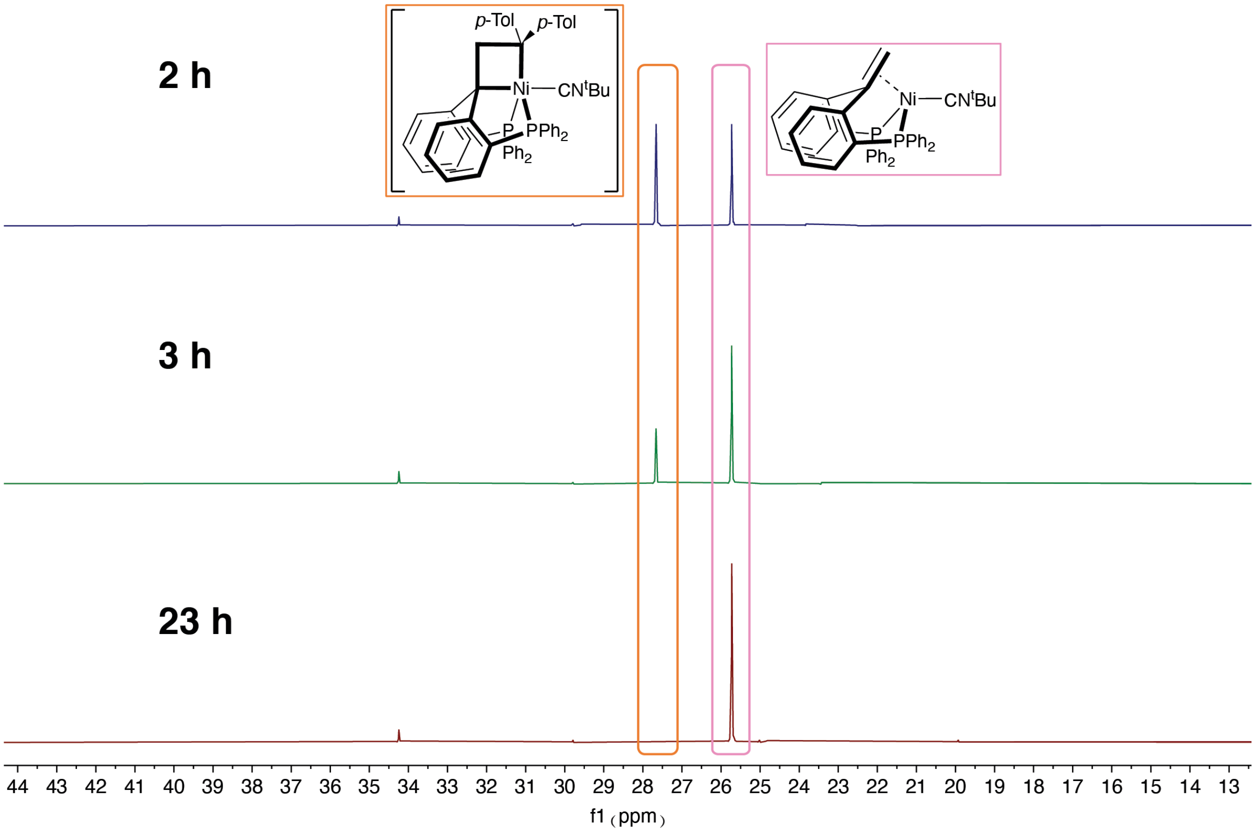

Figure S10. $^{31}P\{^{1}H\}$ NMR spectra in C_6D_6 of the reaction of nickelacyclobutane **1** and two equivalents of *t*-butylisocyanide at different times. The small peak at 34.2 ppm corresponds to an unidentified species that may be associated with the [2+2] cycloreversion process.

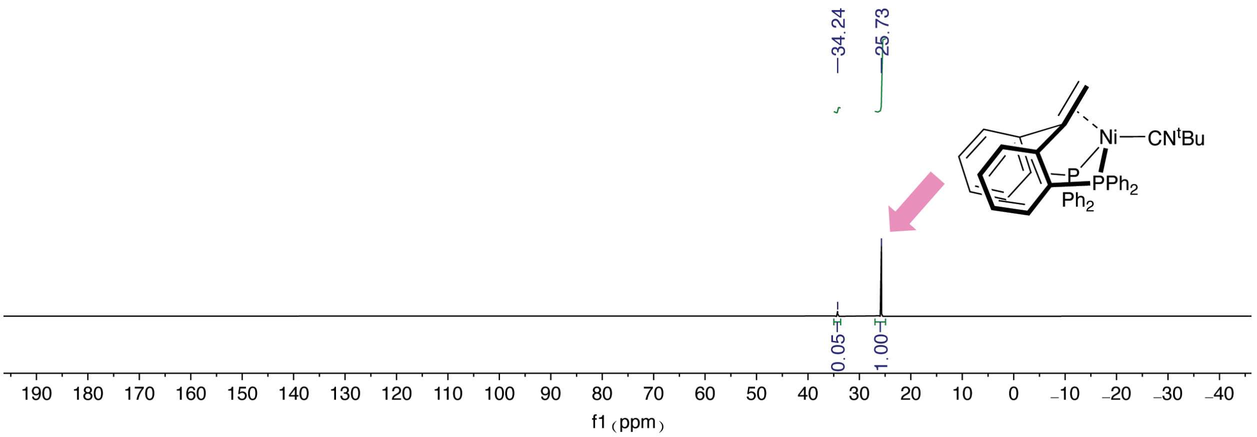

Figure S11. $^{31}P\{^{1}H\}$ NMR spectrum in C_6D_6 of the reaction of nickelacyclobutane **1** and two equivalents of *t*-butylisocyanide after 23 h. The small peak at 34.2 ppm is an unknown species that may be associated with the [2+2] cycloreversion process.

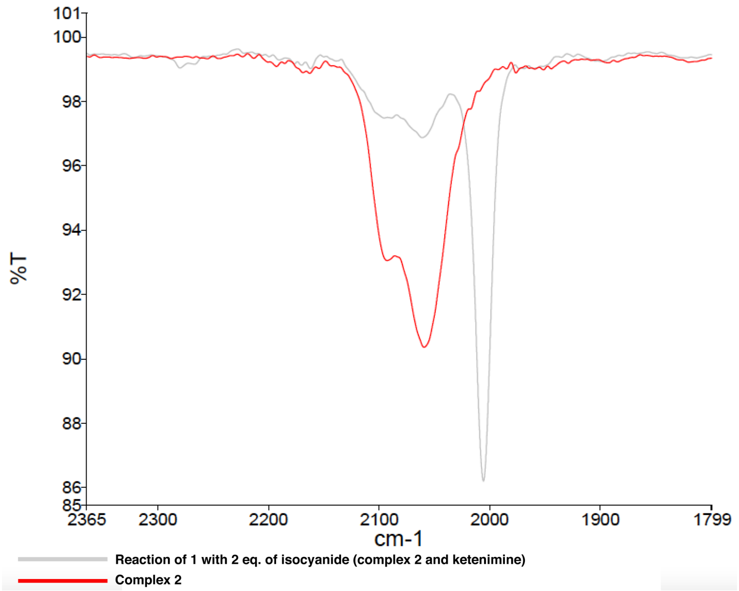

Figure S12. IR spectrum of complex **2** and reaction mixture of nickelacyclobutane **1** + 2 equivalents of CN^tBu.

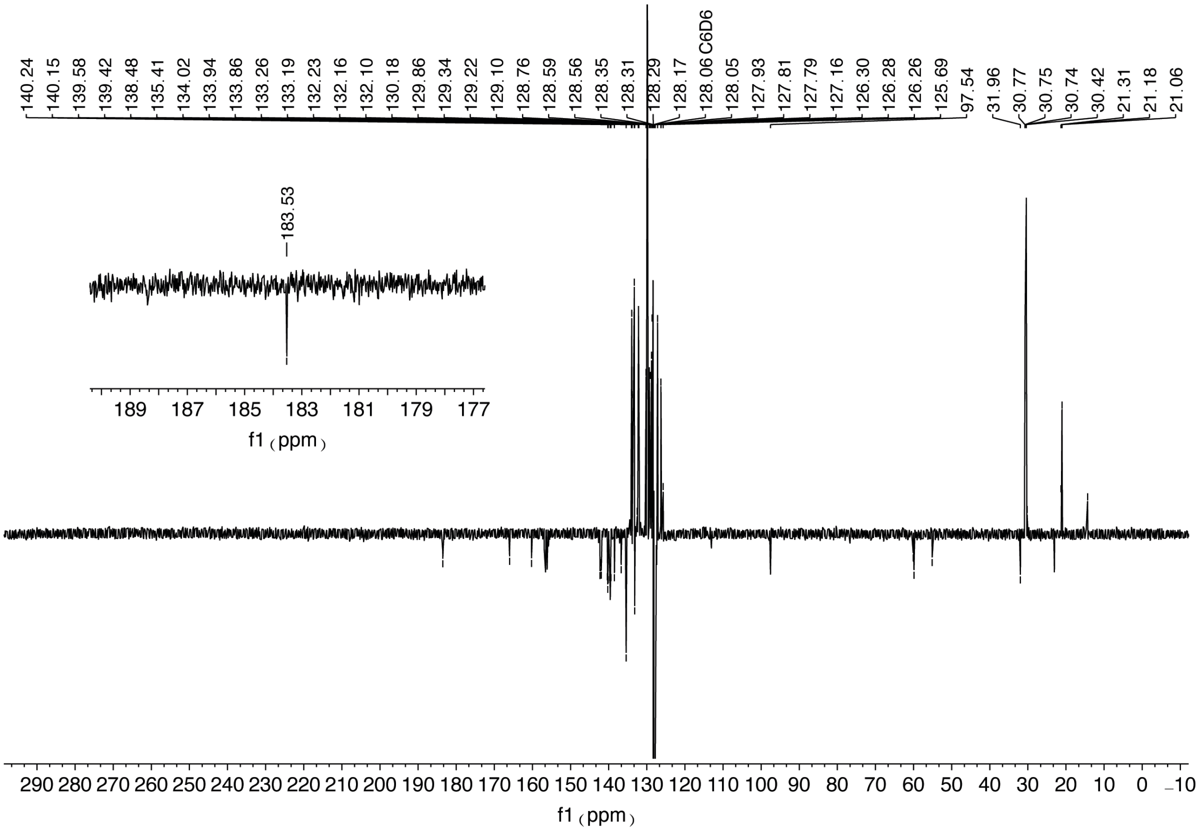

Figure S13. APT $^{13}C\{^1H\}$ in C_6D_6 of the reaction of nickelacyclobutane **1** and two equivalents of *t*-butylisocyanide after 23 h. The peak at 183.5 corresponds to C=*C*=N of the ketenimine.

2.3 Reaction of Nickelacyclobutane (1) and 55 equivalents of *t*-butylisocyanide

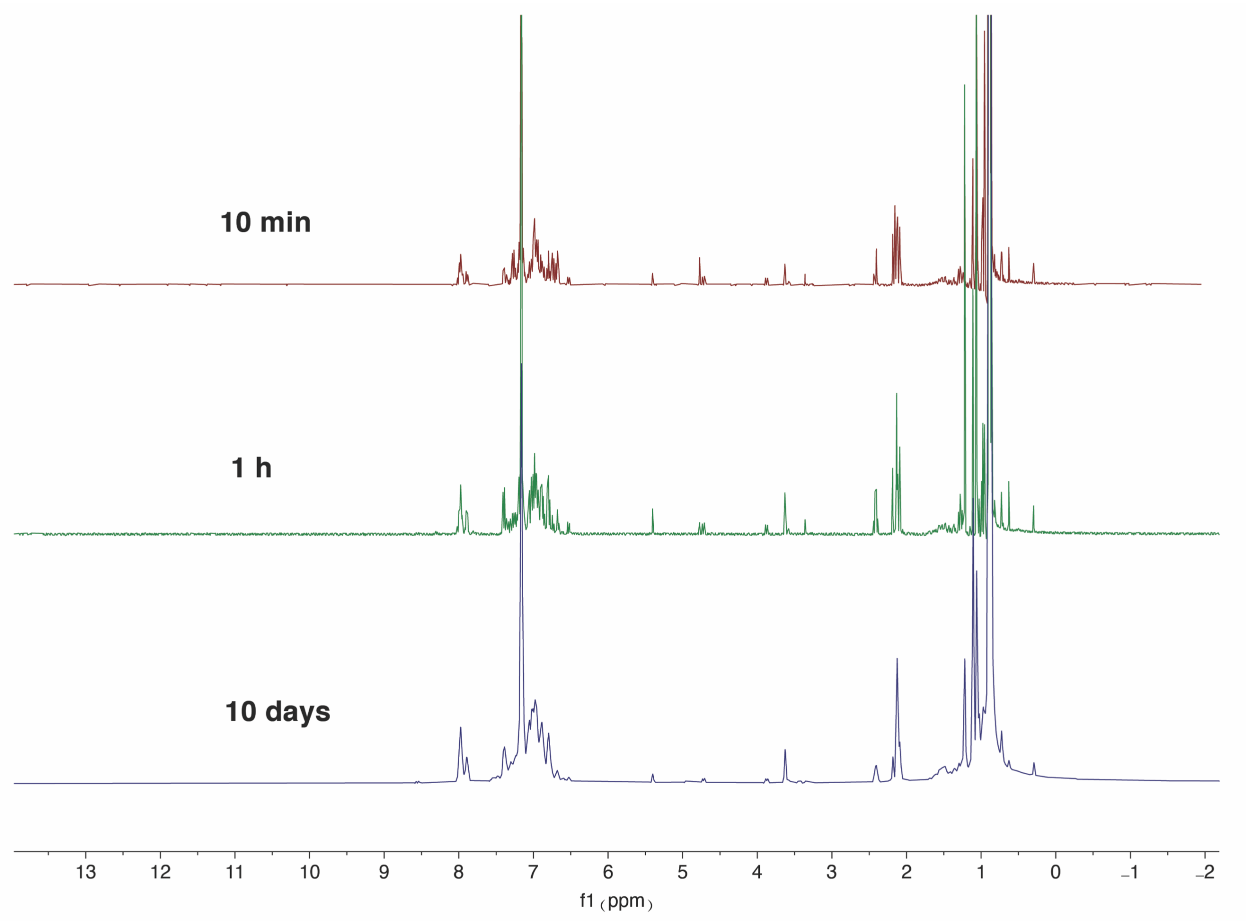

Figure S14. Full ^{1}H NMR spectra (range –2 - 14 ppm) of reaction between 55 equivalents of *t*-butylisocyanide and nickelacyclobutane **1** at different times.

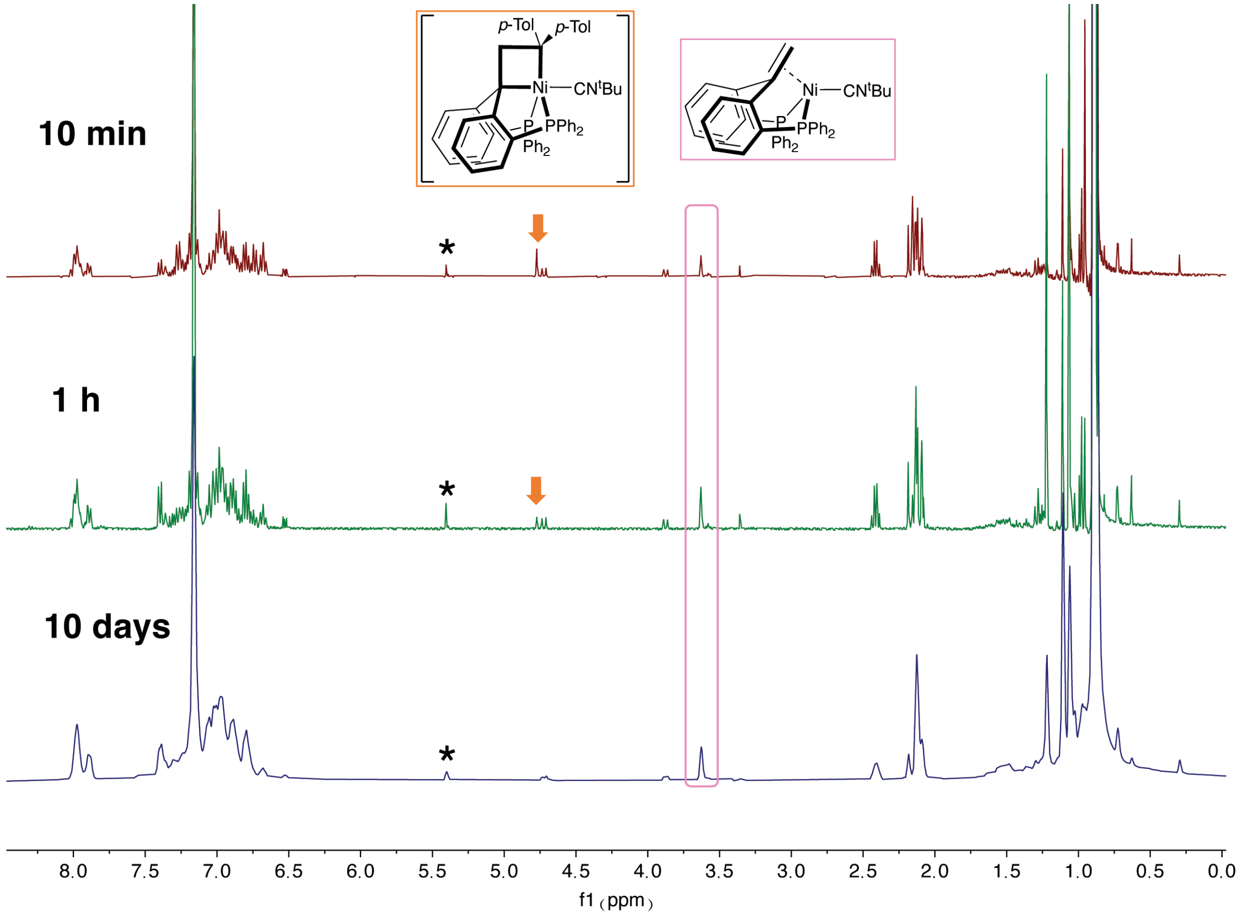

Figure S 15. ^1H NMR spectra in C_6D_6 of reaction between 55 equivalents of *t*-butylisocyanide and nickelacyclobutane **1** at different times (range 0-8.5 ppm). The peak marked with an asterisk correspond to 1,1-di(*p*-tolyl)ethene.

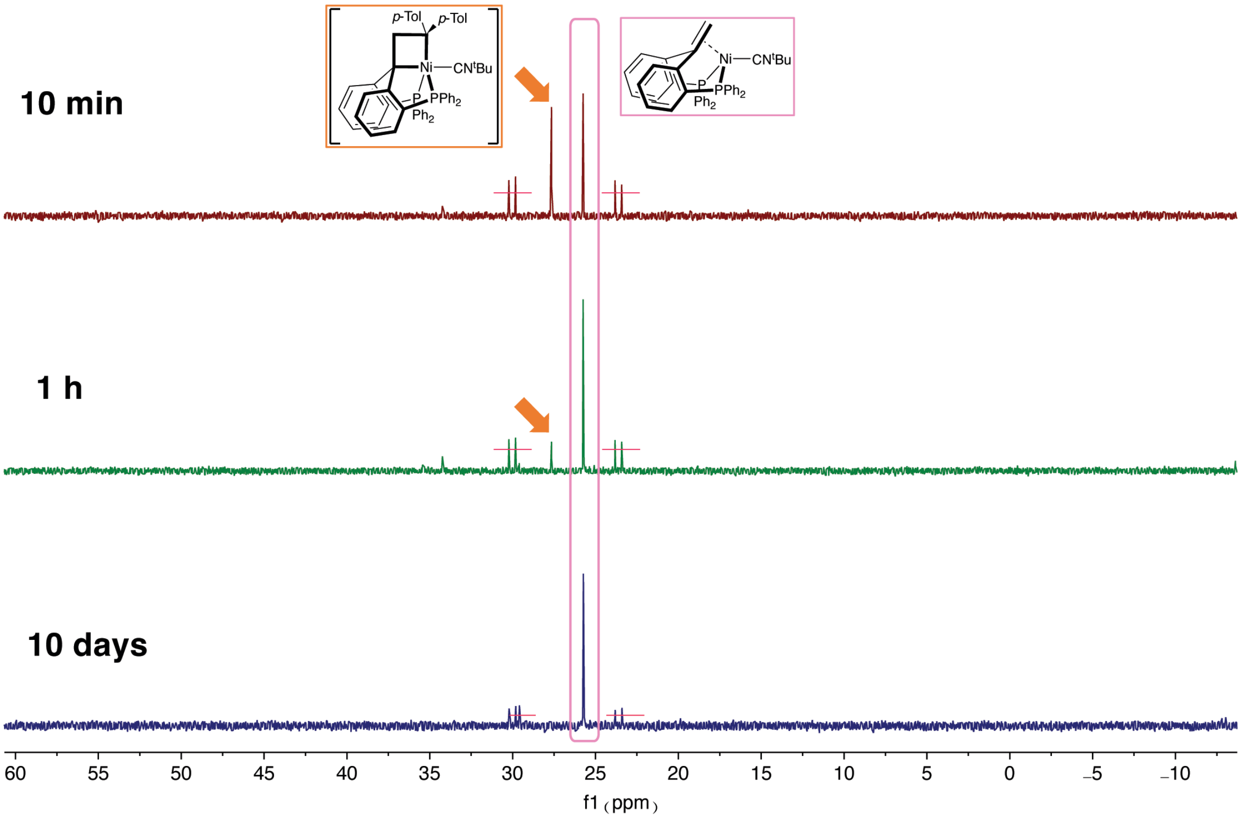

Figure S16. $^{31}P\{^{1}H\}$ NMR in C_6D_6 of the reaction mixture of nickelacyclobutane **1** with 55 equivalents of *t*-butylisocyanide at different times. Crossed doublet peaks (red lines) belong to a small amount of $[^{Ph}dpbe^{H,C(p\text{-}Tol2)}]NiCN^{t}Bu$.

3. Spectra of new compounds

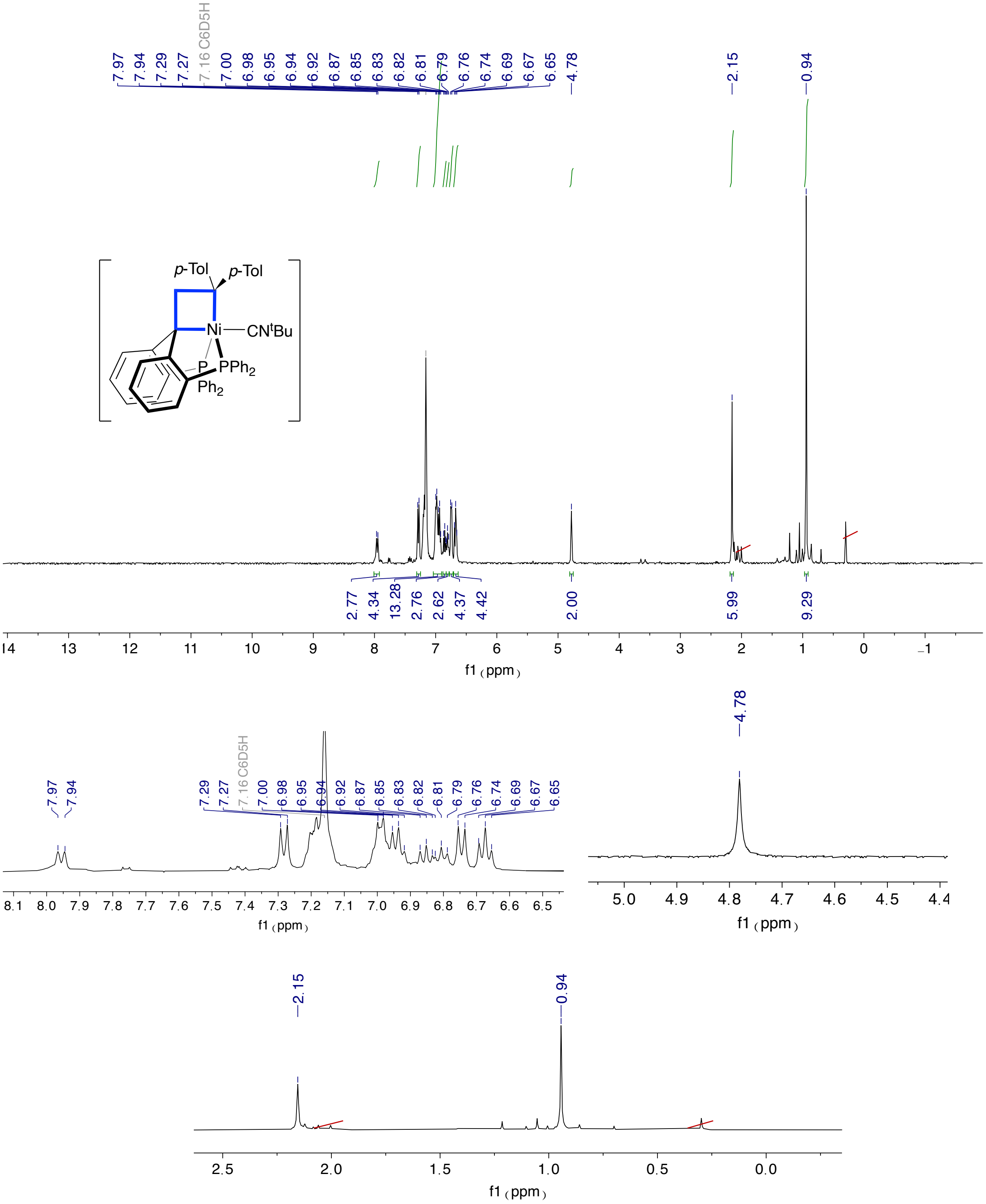

Figure S17. ^{1}H NMR spectrum of **1-CN^{t}Bu** in C_6D_6 at 25 °C. Crossed peaks (red lines) correspond to small amount of di-*p*-tolylketon-azin and silicon grease.

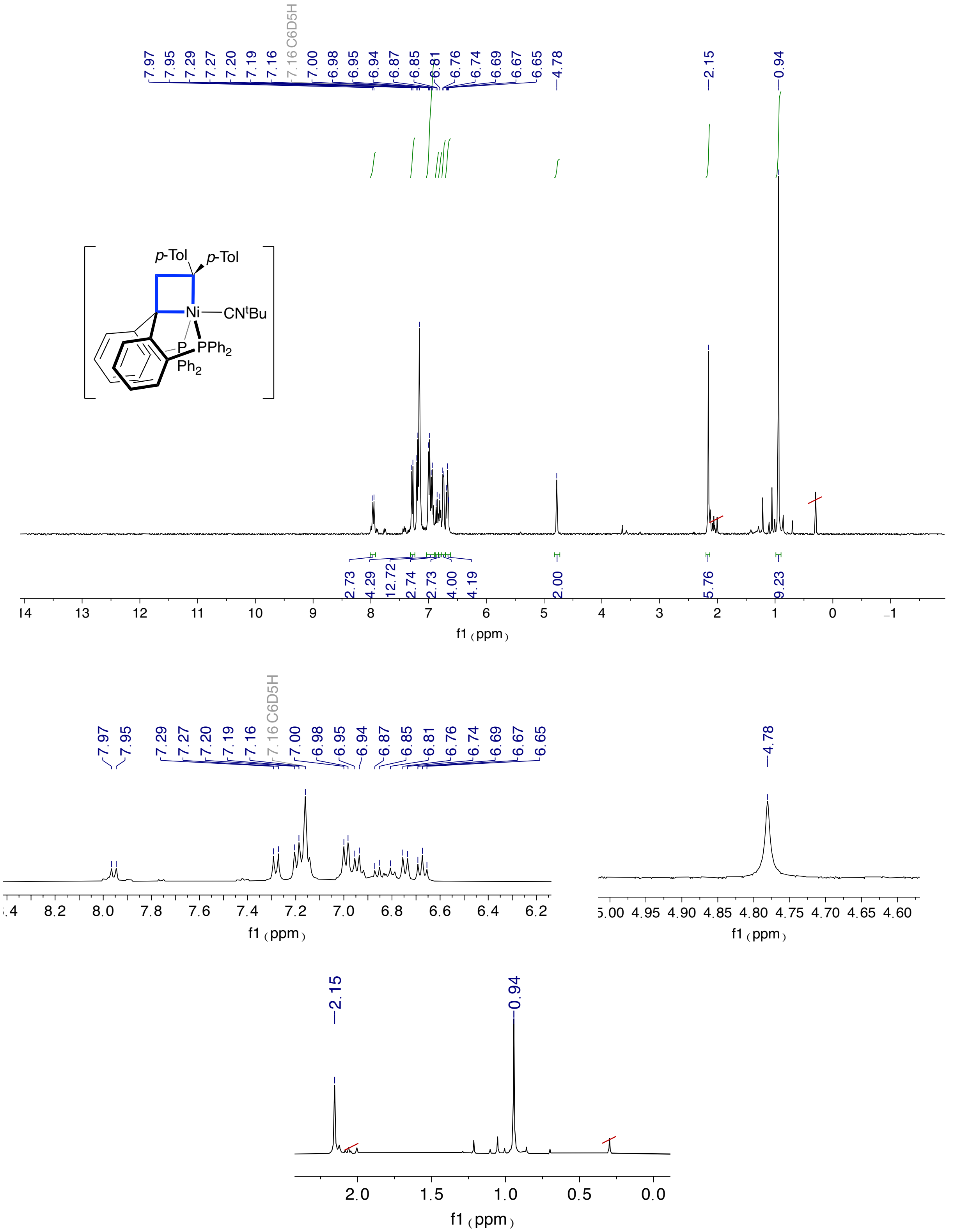

Figure S18. $^{1}H\{^{31}P\}$ NMR spectrum of **1-CN^{t}Bu** in C_6D_6 at 25 ºC. Crossed peaks (red lines) correspond to small amount of di-*p*-tolylketon-azin and silicon grease.

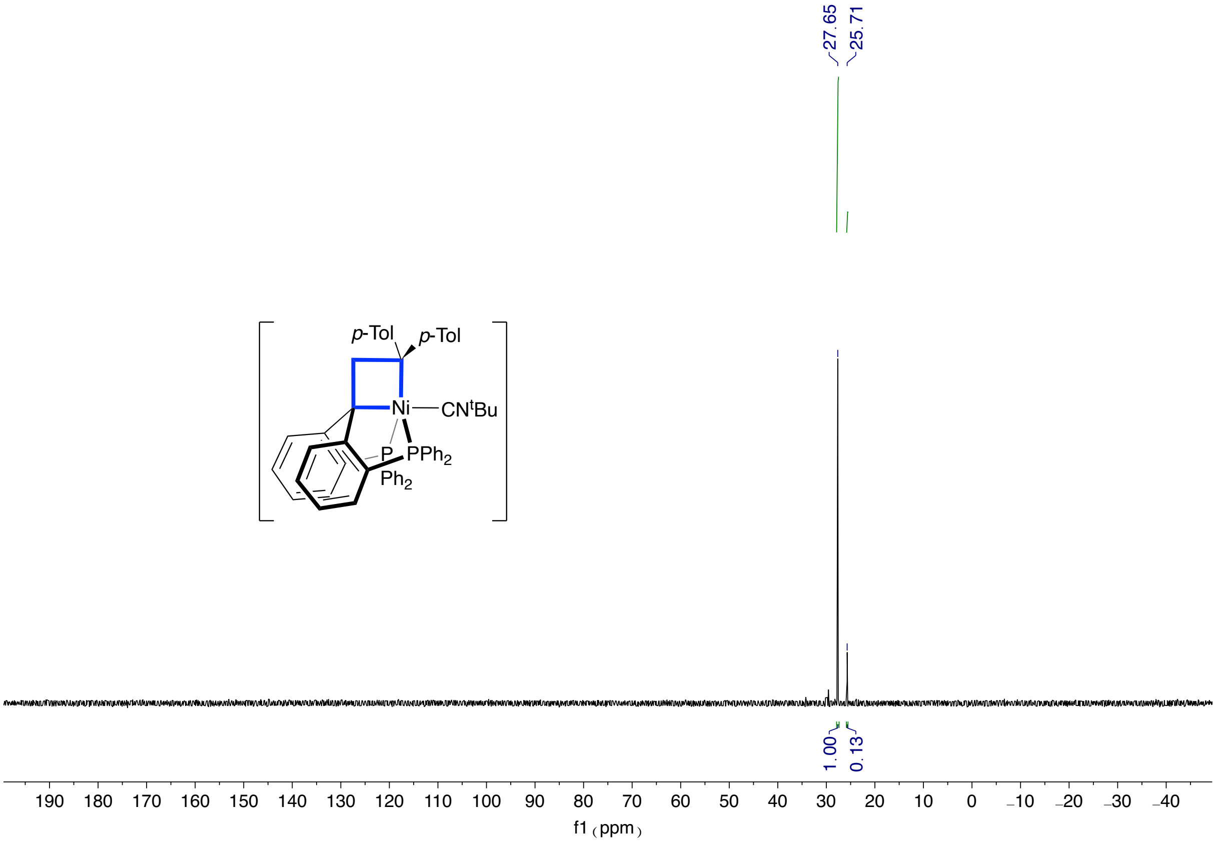

Figure S19. $^{31}P\{^1H\}$ NMR spectrum of **1-CN^{t}Bu** in C_6D_6 at 25 ºC. Small peak at 25.7 ppm correspond to a small amount of complex **2.**

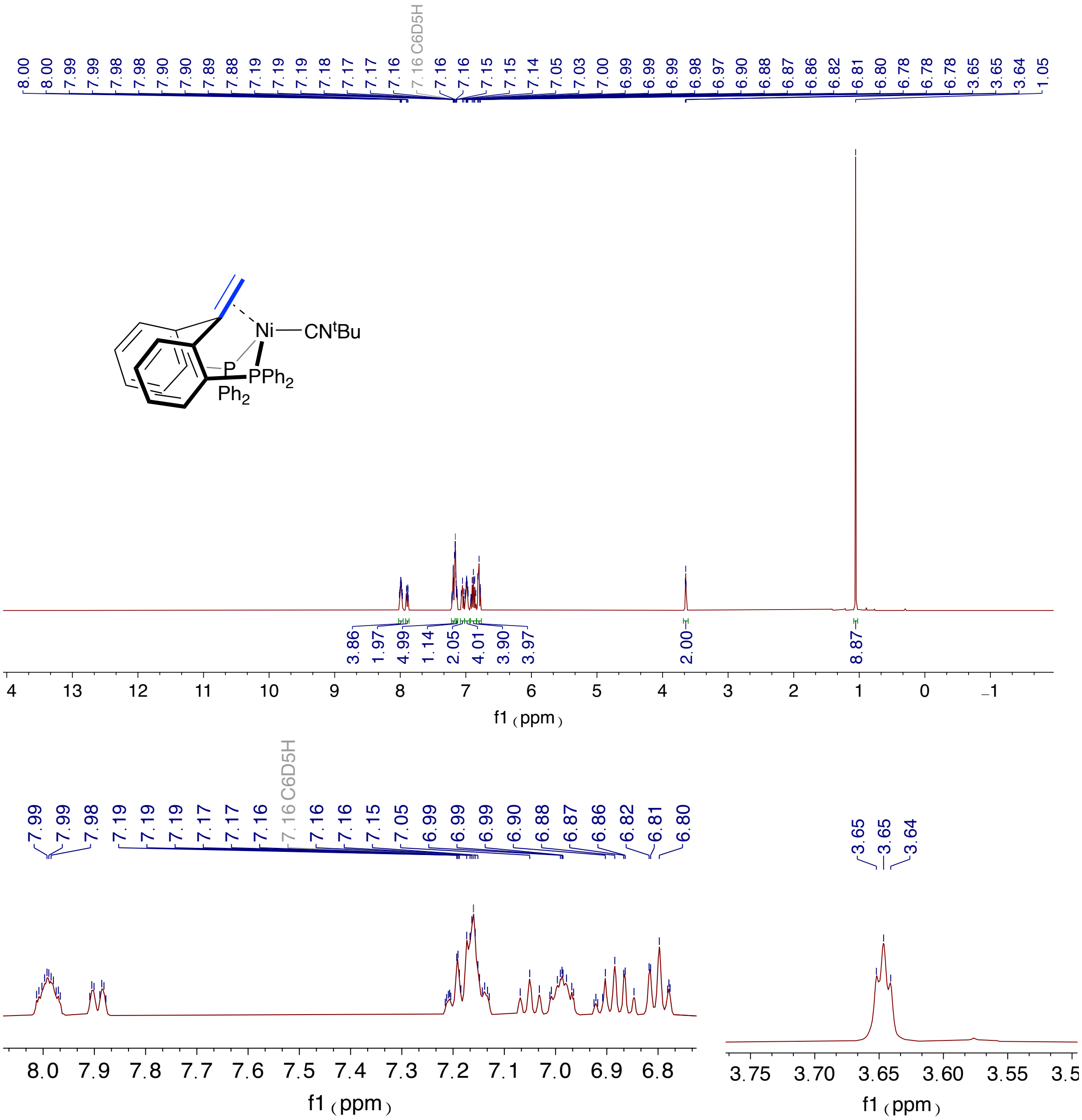

Figure S20. ^{1}H NMR spectrum of complex **2** in C_6D_6 at 25 ºC.

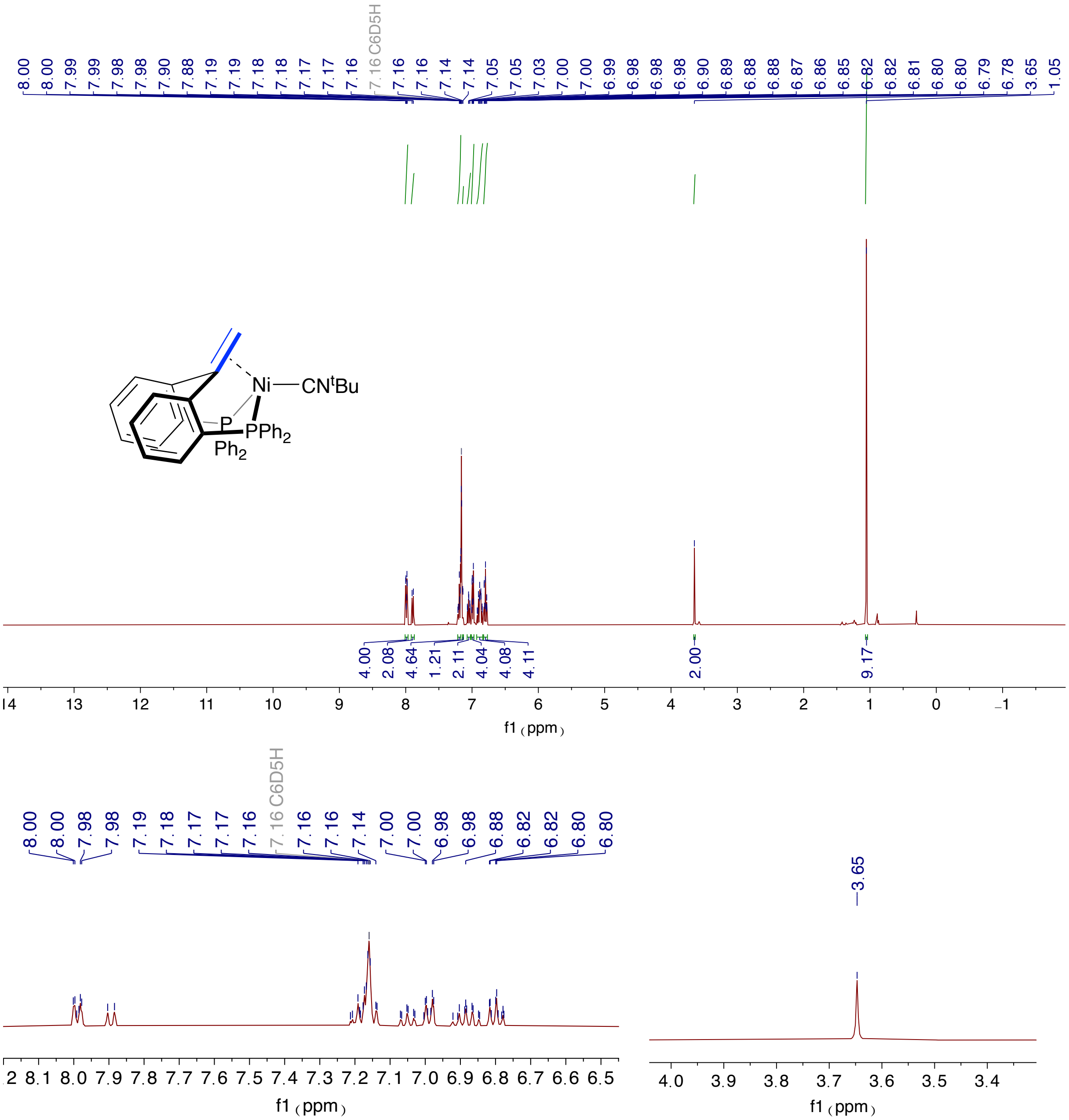

Figure S21. $^1H\{^{31}P\}$ NMR spectrum of complex **2** in C_6D_6 at 25 °C.

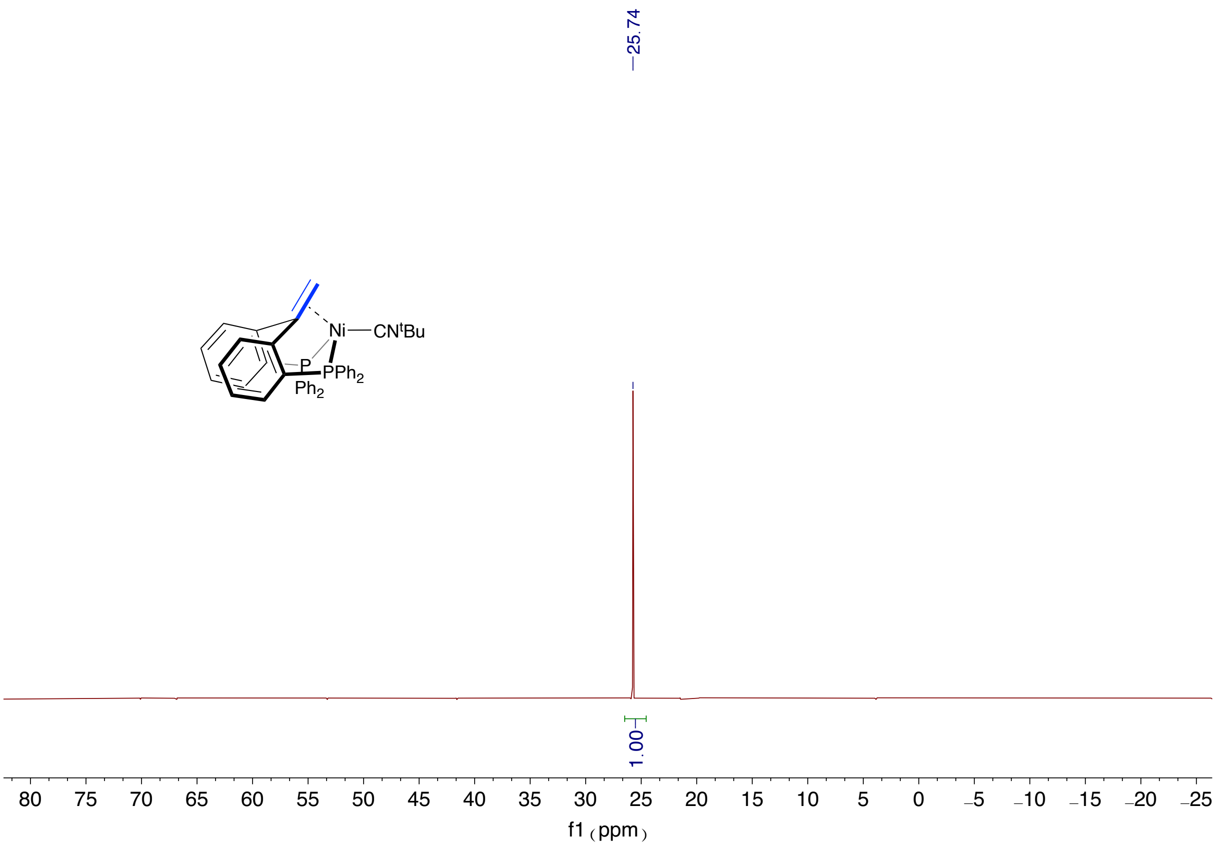

Figure S22. $^{31}P\{^{1}H\}$ NMR spectrum of complex **2** in C_6D_6 at 25 °C.

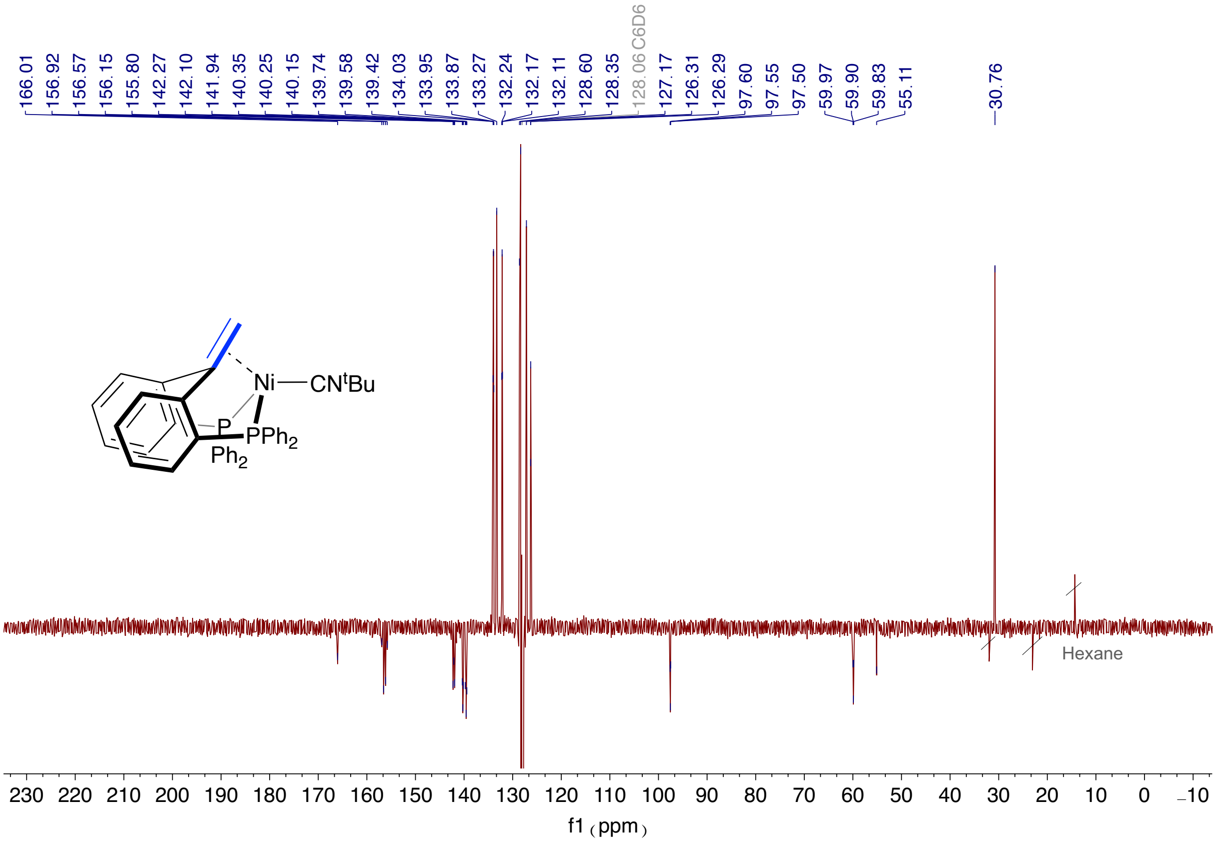

Figure S23. ^{13}C APT of complex **2** in C_6D_6 at 25 °C.

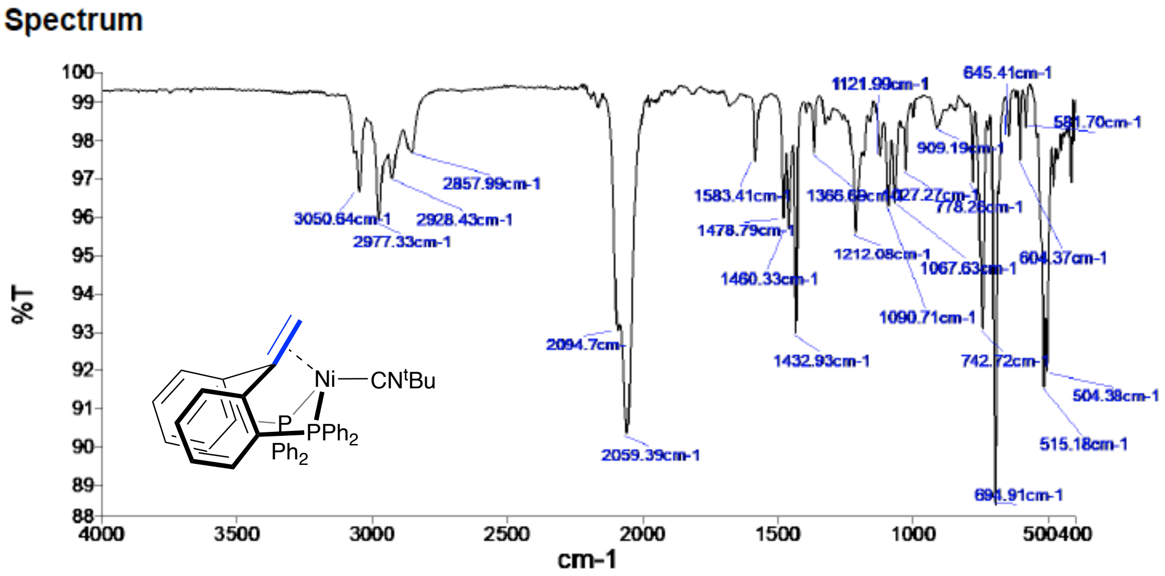

Figure S24. FT-IR spectrum of complex **2**.

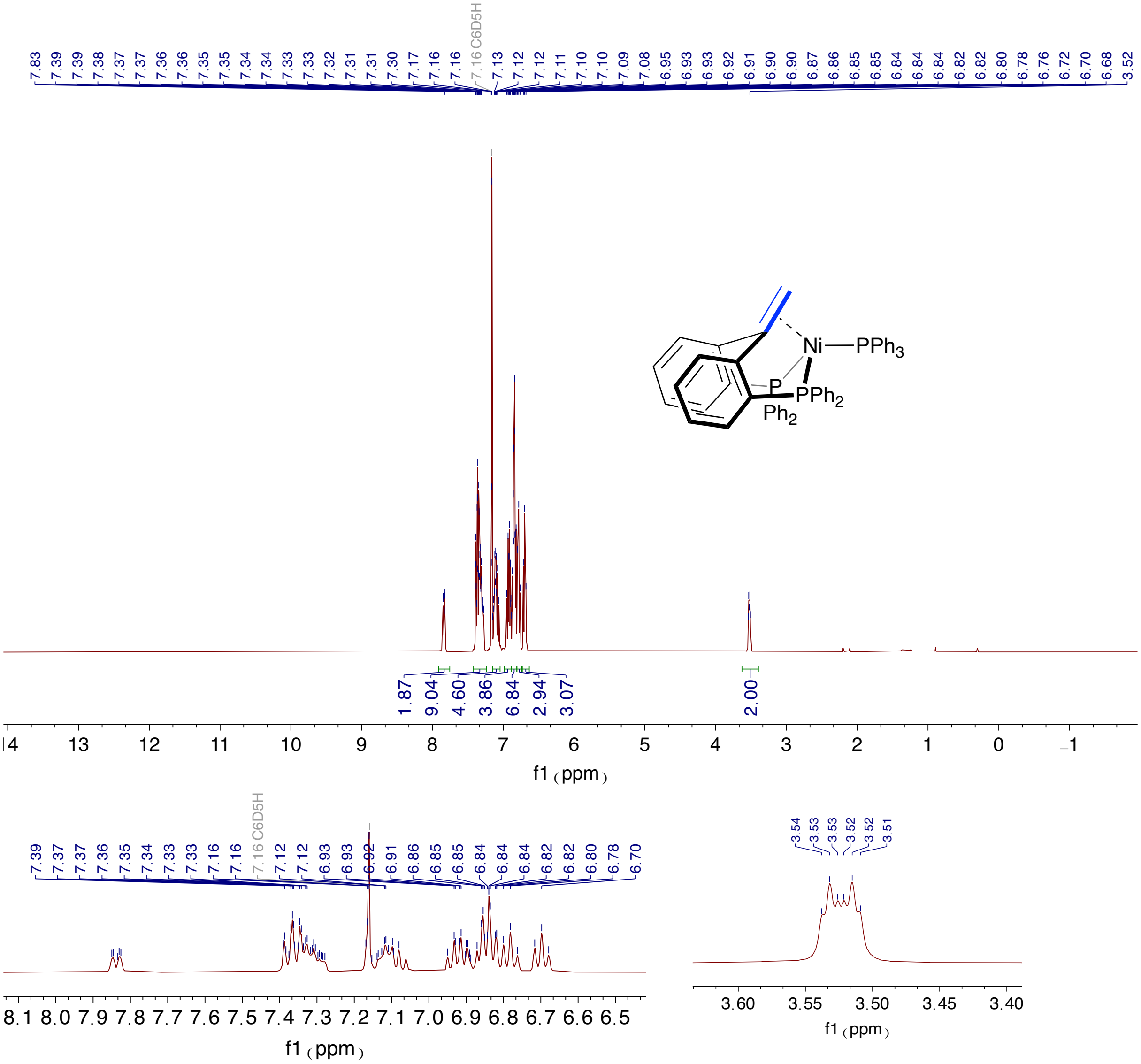

Figure S25. ^1H NMR spectrum of complex **3** in C_6D_6 at 25 ºC.

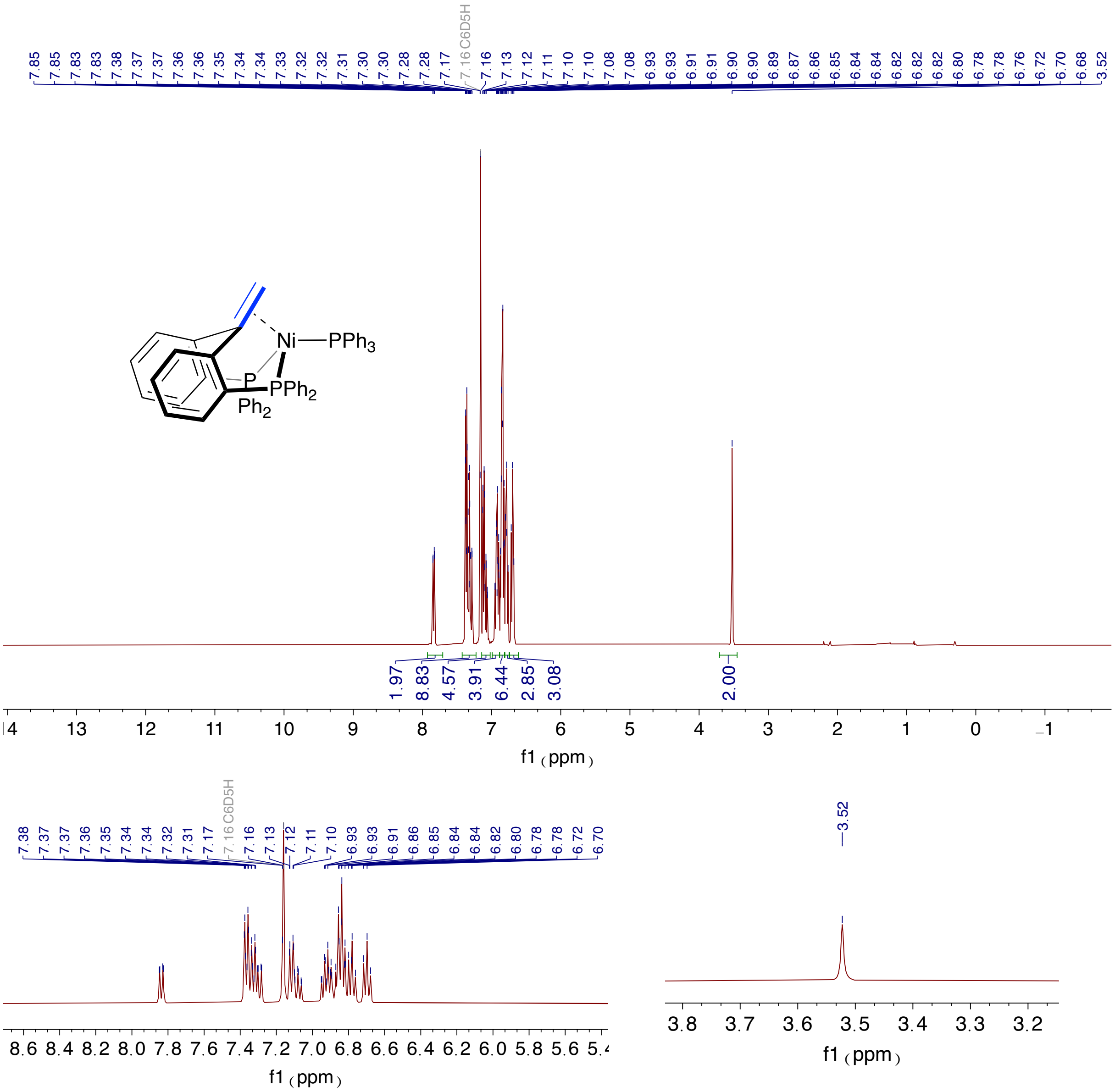

Figure S26. $^{1}H\{^{31}P\}$ NMR spectrum of complex **3** in C_6D_6 at 25 ºC.

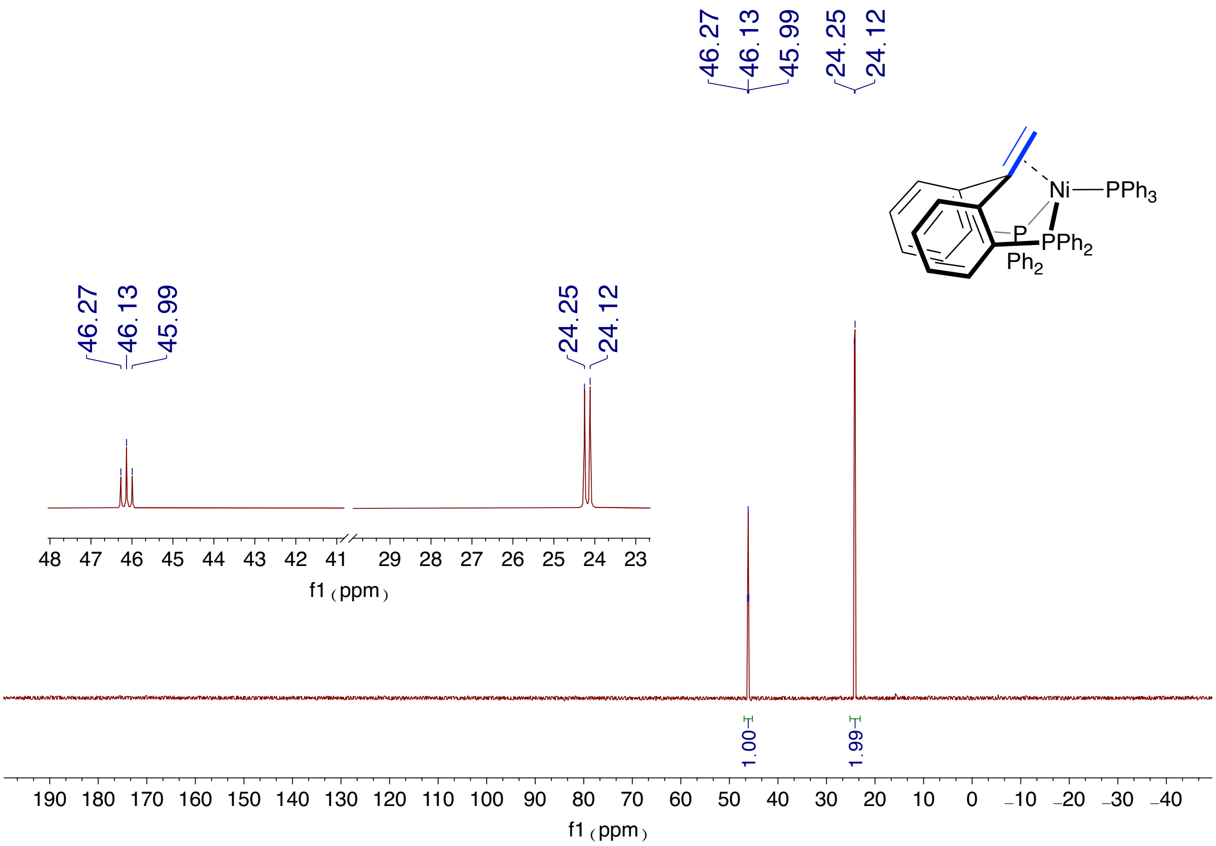

Figure S27. $^{31}P\{^{1}H\}$ NMR spectrum of complex **3** in C_6D_6 at 25 °C.

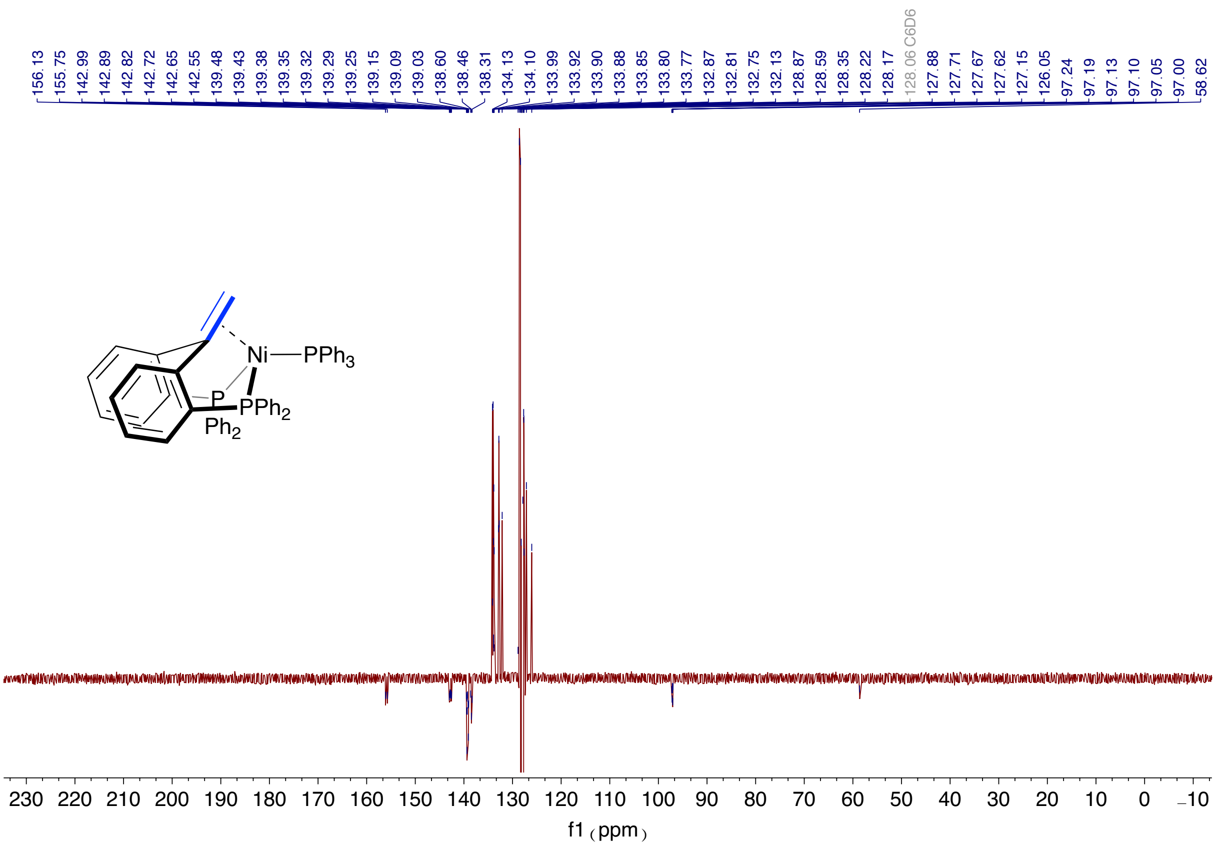

Figure S28. ^{13}C APT of complex **3** in C_6D_6 at 25 °C.

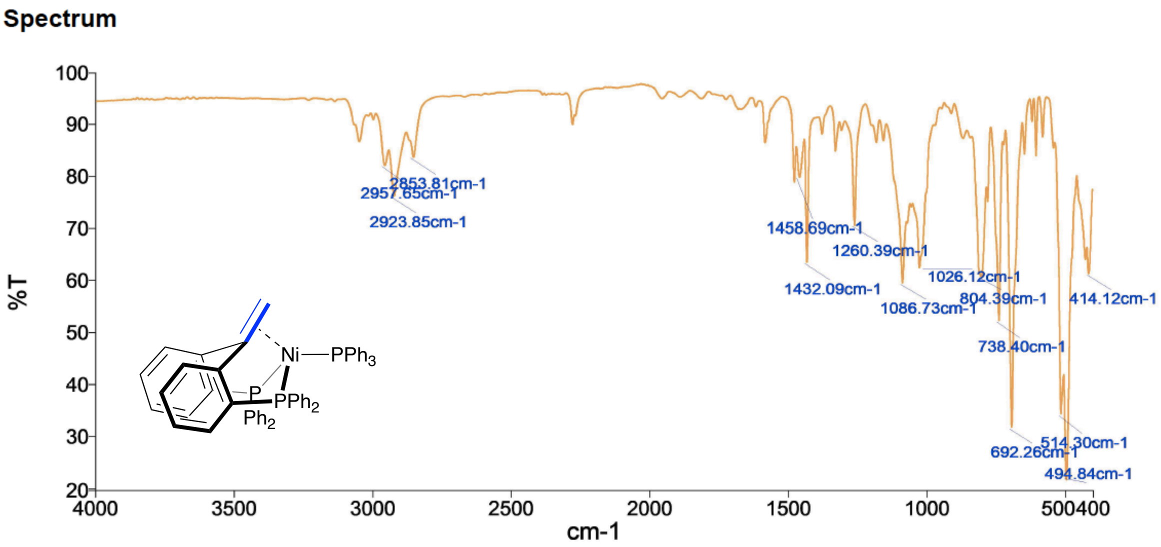

Figure S29. FT-IR spectrum of complex **3**.

4. X-ray crystal structure determination

(^{Ph}bppe^{H,H})Ni(PPh$_3$) (**4**): $C_{56}H_{45}NiP_3$ + disordered solvent, Fw = 869.54[*], orange needle, 0.42 × 0.10 × 0.05 mm^3, monoclinic, $P2_1/n$ (no. 14), a = 9.5744(4), b = 23.4453(7), c = 21.3424(11) Å, β = 100.245(2)°, V = 4714.5(3) Å^3, Z = 4, D_x = 1.225 g/cm^3[*], μ = 0.55 mm^{-1}[*]. The diffraction experiment was performed on a Bruker Kappa ApexII diffractometer with sealed tube and Triumph monochromator (λ = 0.71073 Å) at a temperature of 150(2) K up to a resolution of $(\sin\theta/\lambda)_{max}$ = 0.65 Å^{-1}. The crystal appeared to be cracked into two fragments. Consequently, two orientation matrices were used for the intensity integration with the Eval15 software[2] resulting in a HKLF5-file.[3] A multi-scan absorption correction and scaling was performed with TWINABS[4] (correction range 0.48-0.75). A total of 90775 reflections was measured, 12991 reflections were unique (R_{int} = 0.131), 7764 reflections were observed [$I>2\sigma(I)$]. The structure was solved with Patterson superposition methods using SHELXT.[5] Structure refinement was performed with SHELXL-2018[6] on F^2 of all reflections. The crystal structure contains voids (545 Å^3 / unit cell) filled with disordered solvent molecules. Their contribution to the structure factors was secured by back-Fourier transformation using the SQUEEZE algorithm[7] resulting in 103 electrons / unit cell. Non-hydrogen atoms were refined freely with anisotropic displacement parameters. Hydrogen atoms were introduced in calculated positions and refined with a riding model. 550 Parameters were refined with no restraints. R1/wR2 [$I > 2\sigma(I)$]: 0.0669 / 0.1318. R1/wR2 [all refl.]: 0.1360 / 0.1558. S = 1.018. Residual electron density between -0.51 and 0.58 e/Å^3. Batch scale factor for the second crystal fragment BASF = 0.1885(13). Geometry calculations and checking for higher symmetry was performed with the PLATON program.[8]

[*] Derived values do not contain the contribution of the disordered solvent.

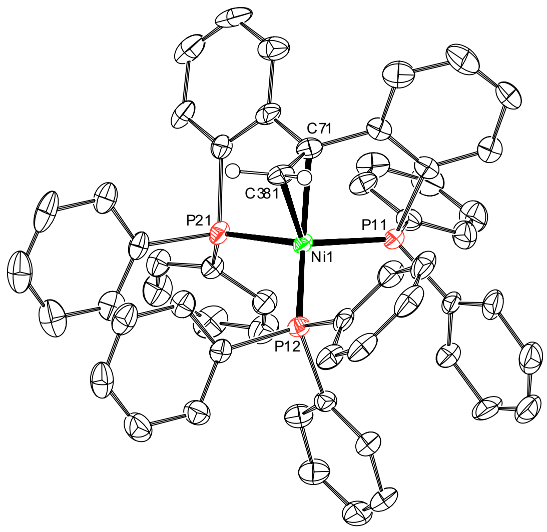

Figure S30. Molecular structure of complex **4**. Displacement ellipsoids are drawn at the 50% probability level. Solvent molecules, most H atoms are omitted for clarity. Selected bond lengths: (Å): C71–C381 1.400(6), C71–NI1 2.024(4), C381–NI1 2.048(4), NI1–P12 2.1607(11), NI1–P11 2.1669(11), NI1–P21 2.1818(11).

5. DFT studies

5.1 General information

DFT calculations were performed using the Gaussian 16 software package version C.01.[9] Geometry optimizations were carried out in vacuum at the B3LYP-GD3BJ/6-31g(d,p) level of theory on all atoms. Frequency analyses on all stationary points were used to ensure that they are minima (no imaginary frequency) or transition states (one imaginary frequency). Transition states were calculated using the QST3 (synchronous transit-guided quasi-Newton number 3) method or using the opt=TS (Berny algorithm) keyword. The guess structure proposed for each TS calculation was based on the results of relaxed potential energy surface scans (PES). The transition state was validated by geometry optimization of the transition state towards reactant and product, for the direct carbene transfer transition states (TS 5 and 16) intrinsic reaction coordinated (IRC) scan along the imaginary vibrational frequency was performed. ΔG° was calculated by single point calculation at B3LYP-GD3BJ/def2TZVP(C_6H_6) level of theory with the Solvation Model Base on Density (SMD) adjusting the value with the thermal correction obtained at the B3LYP-GD3BJ/6-31g(d,p) level of theory with temperature 298.15 K and pressure 1 atmosphere.

5.2 Isomers of complex 6

Several isomers of **6** were calculated showing similar energy (Figure S31). The isomer where the ketenimine is coordinated in η^1(N) showed the lowest energy (–1.9 kcal/mol). Nevertheless, the π-complex **6** as formed after carbene transfer/reductive fragmentation is shown in most figures for clarity.

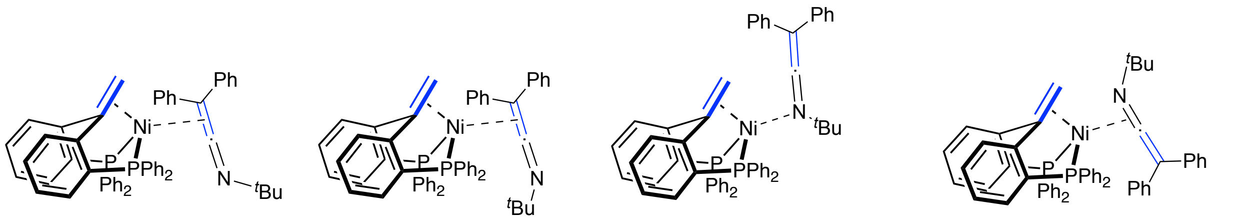

6 **-1.2 kcal/mol** | **6-1** **5.9 kcal/mol** | **6- η^1(N)** **-1.9 kcal/mol** | **6- η^2(C,N)** **1.8 kcal/mol**

Figure S31. Gibbs energy of the isomers of **6** calculated at B3LYP-GD3BJ/def2TZVP/SMD(C_6H_6)//B3LYP-GD3BJ/6-31g(d,p) level of theory. Additional information of 1,1-insertion route

5.2.1 1,1-insertion route towards other nickelacyclopentane isomer

Two geometric isomers of nickelacyclopentane can be obtained. We described in the main text the formation of complex **5** with the lone pair of the nitrogen atom pointing opposite to the nickel center (Figure 2). Insertion to form a stereoisomeric nickelacyclopentane with the nitrogen lone pair pointing towards nickel (**10**) is energetically forbidden with an overall barrier of 35.2 kcal/mol (Figure S32).

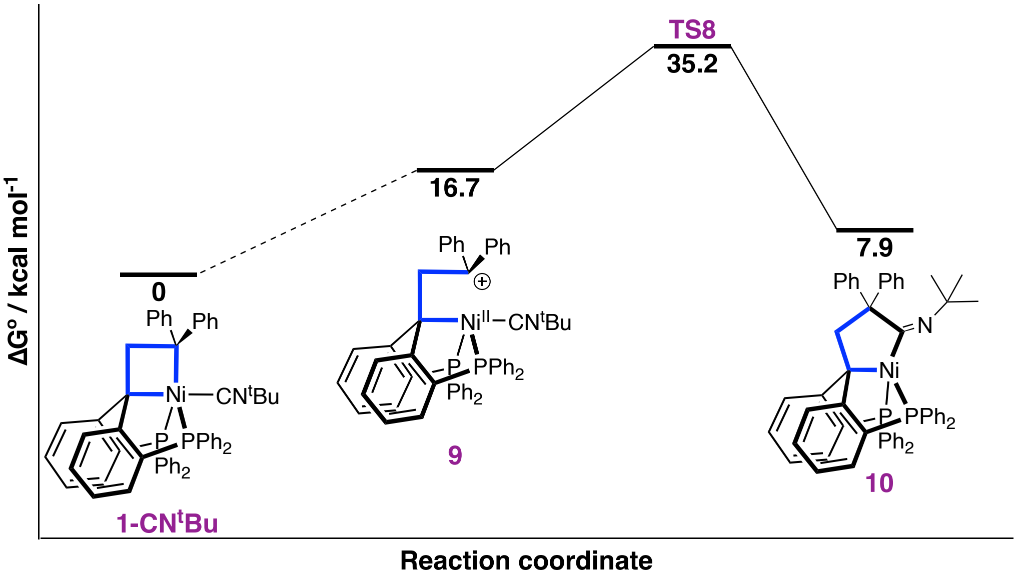

Figure S32. Gibbs free energy profile for formation of **10** (lone pair towards nickel) computed at the B3LYP-GD3BJ/def2TZVP/SMD(C_6H_6)//B3LYP-GD3BJ/6-31g(d,p) level of theory. Dashed lines connect intermediates between which no transition state was optimize

5.2.2 1,1-insertion route involving two molecules of *t*-butylisocyanide

We considered the effect of having an excess of isocyanide for the formation of the ketenimine product (Figure S33). Coordination of an additional molecule of isocyanide to complex **5** is accessible but strongly endergonic ($\Delta G^{\ddagger}$= 21.3 kcal/mol). Two routes for the cleavage of the nickelacyclopentane to yield the product were found, a [3+2] cycloreversion process yielding complex 2 and the ketenimine product ($\Delta G^{\ddagger}$= 50.9 kcal/mol) and a cycloreversion pathway involving the decoordination of one phosphine arm ($\Delta G^{\ddagger}$= 46.7 kcal/mol). Both processes are prohibitively high in energy and non-feasible under the reaction conditions.

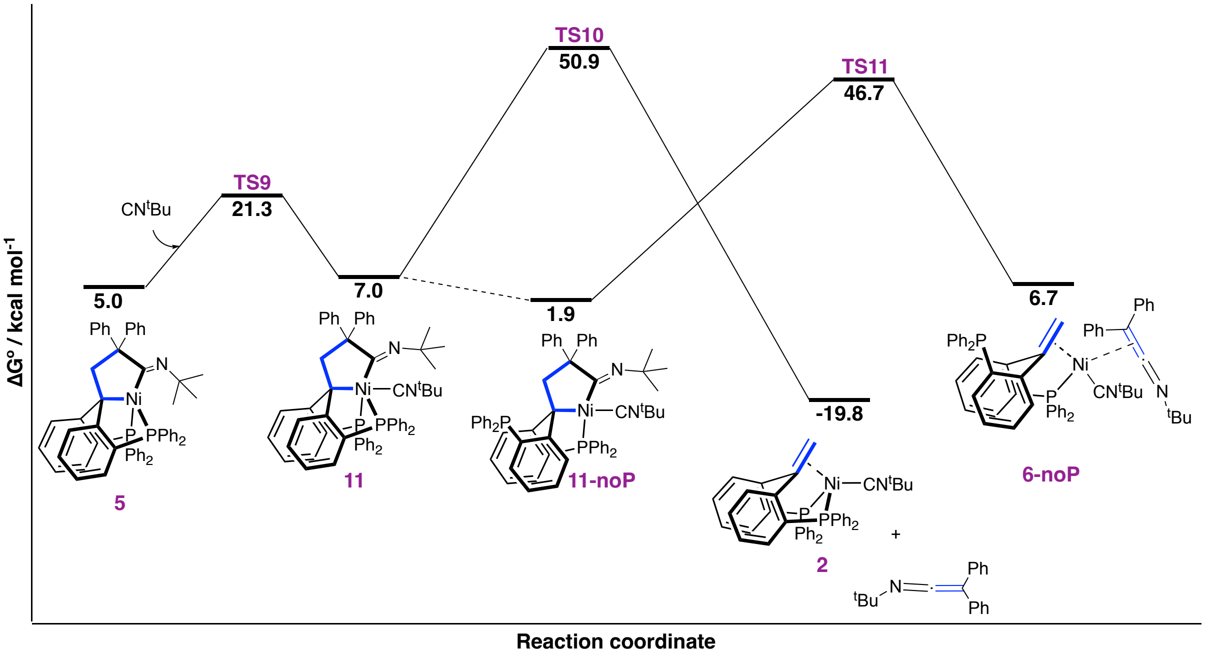

Figure S33. Gibbs free energy profile for formation of ketenimine from complex **15** computed at the B3LYP-GD3BJ/def2TZVP/SMD(C_6H_6)//B3LYP-GD3BJ/6-31g(d,p) level of theory. Dashed lines connect intermediates between which no transition state was optimized.

5.2.3 1,1-insertion route towards in triplet state

1,1-insertion process involving triplet state nickelacyclopentane intermediates was considered (Figure S34), but the overall barriers for the formation of these intermediates was unfeasible (ΔG‡= 29.9 and 31.3 kcal/mol).

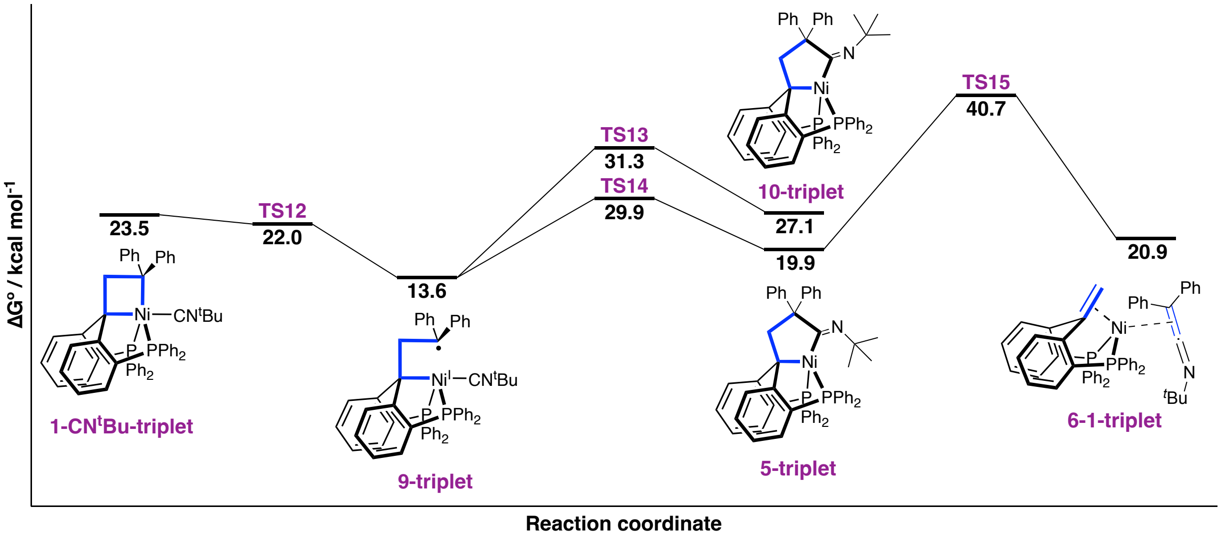

Figure S34. Gibbs free energy profile for formation of ketenimine via 1,1-insertion in triplet state. Computed at the B3LYP-GD3BJ/def2TZVP/SMD(C_6H_6)//B3LYP-GD3BJ/6-31g(d,p) level of theory. Computed energy of **1-CN^{t}Bu-triplet** is slightly higher than **TS12**, this could be a consequence of the different optimization method used for the transition state.

5.3 Additional information of [2+2] Cycloreversion route

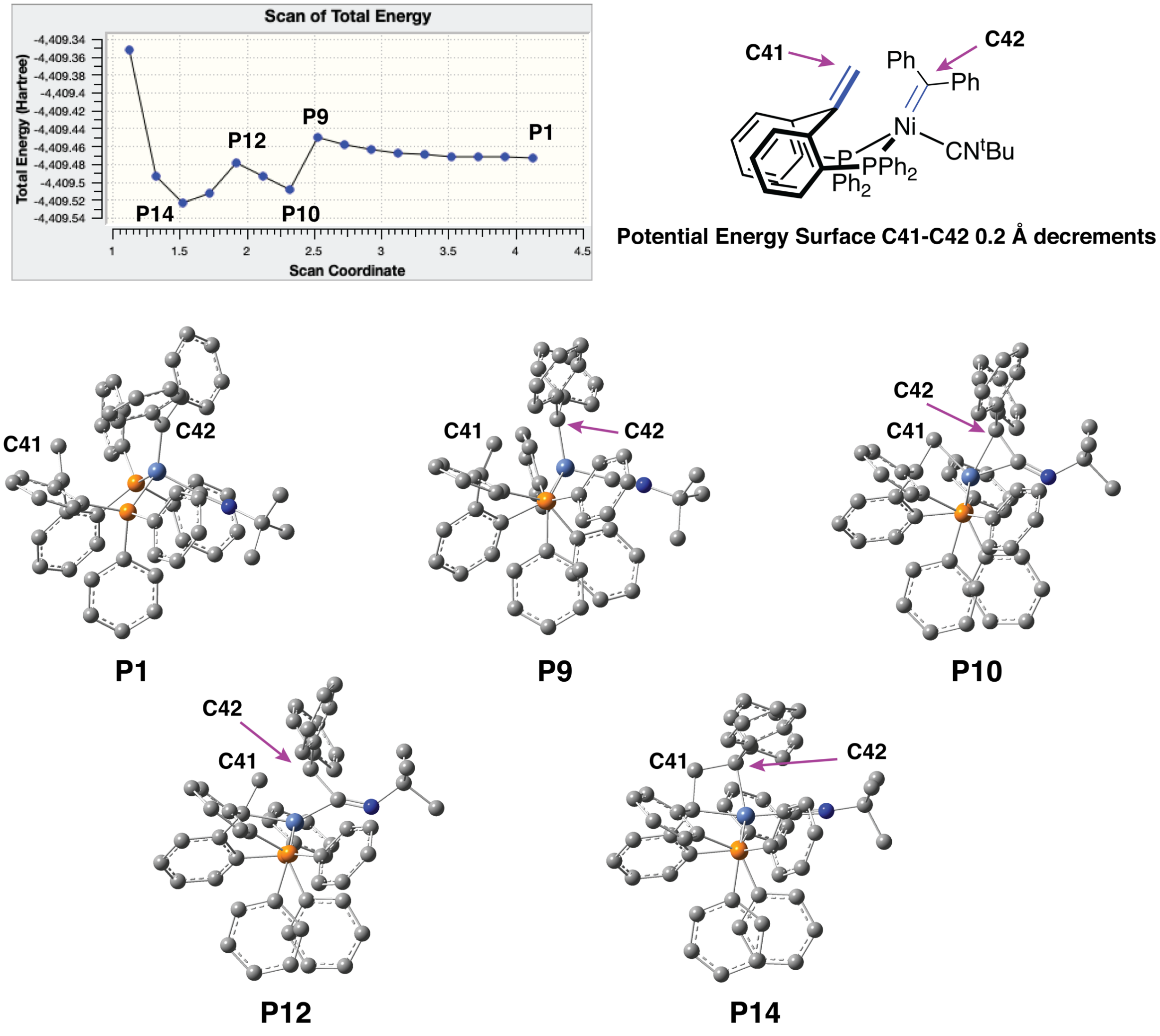

Figure S35. Potential energy scan along the distance between C41 and C42 from complex **8**.

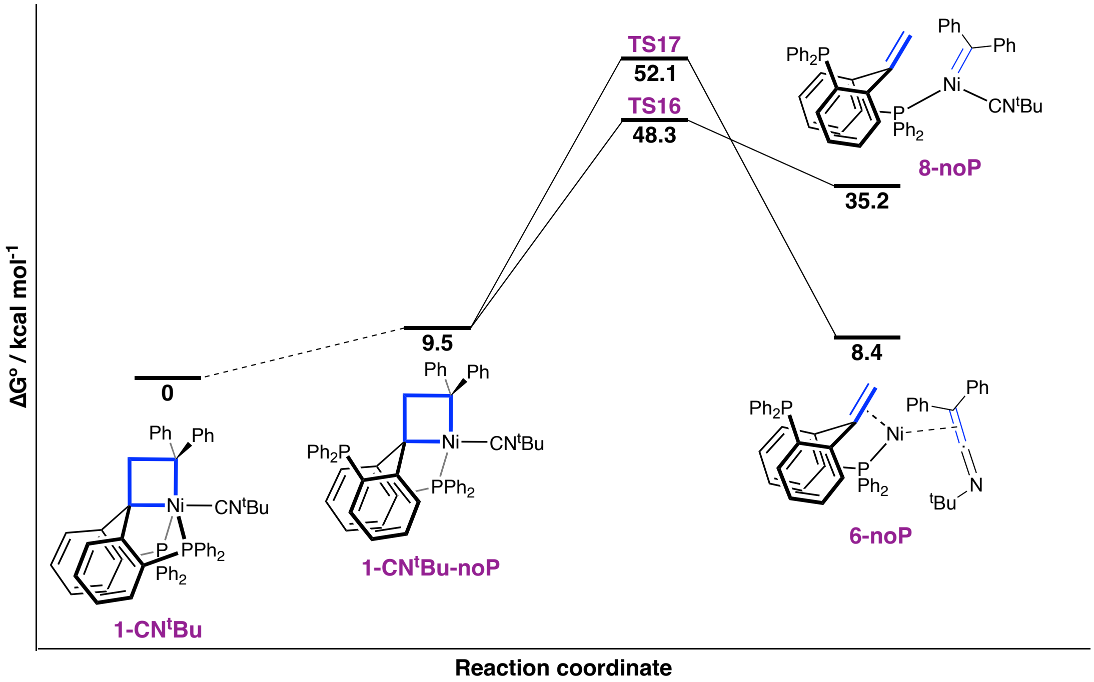

Figure S36. Direct carbene transfer and [2+2] cycloreversion route from **1**-CN^{t}Bu-noP computed at the B3LYP-GD3BJ/def2TZVP/SMD(C_6H_6)//B3LYP-GD3BJ/6-31g(d,p) level of theory. Dashed lines connect intermediates between which no transition state was optimized.

5.4 Additional 1-CN^{t}Bu routes

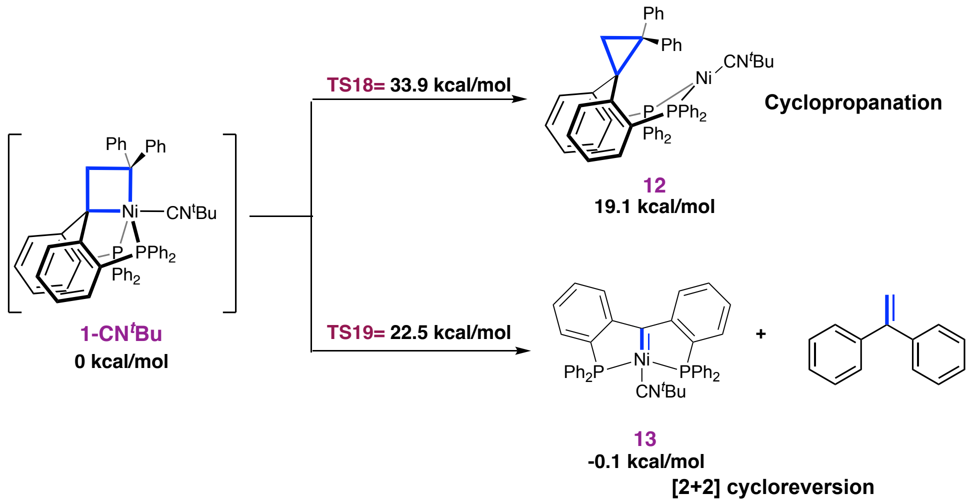

Figure S37. Other competitive fragmentation routes of **1-CN^{t}Bu** and their respective Gibbs free energies computed at the B3LYP-GD3BJ/def2TZVP/SMD(C_6H_6)//B3LYP-GD3BJ/6-31g(d,p) level of theory.

5.5 Comparison with 1-CO

We previously reported the decomposition of the pentacoordinated nickelacyclobutane with CO.[1] The slight differences in comparison with the values previously reported come from two minor changes in the computational methods: the tolyl groups have been replaced by phenyl groups, and the effect of the solvent was added by Solvation Model Density (SMD) using benzene.

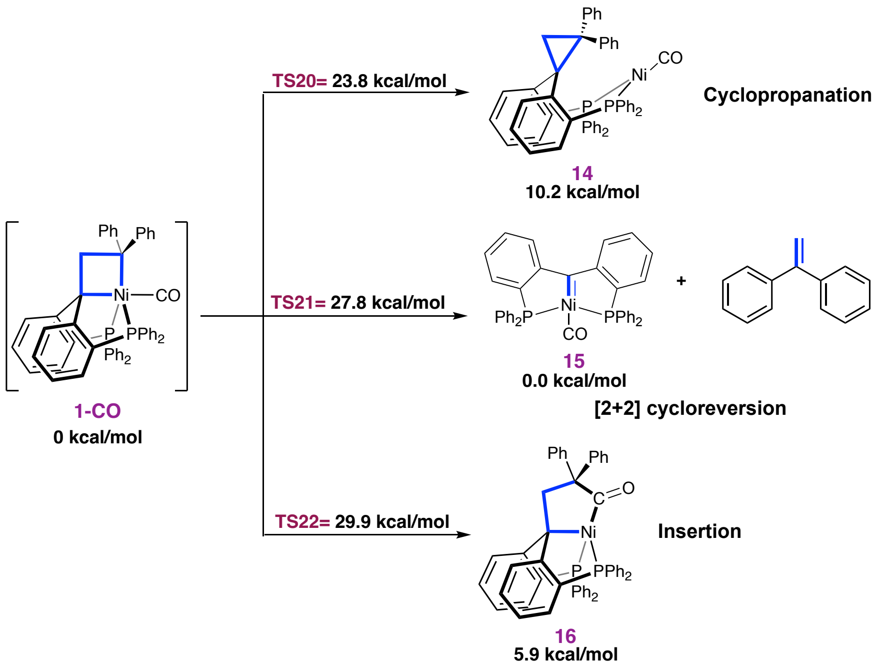

Figure S38. Computed routes of decomposition of **1-CO** and their respective Gibbs free energies, computed at the B3LYP-GD3BJ/def2TZVP/SMD(C_6H_6)//B3LYP-GD3BJ/6-31g(d,p) level of theory.

5.6 Table of energies

Table S1. Single point energies obtained at the B3LYP-GD3BJ/DEF2TZVP/SMD(C_6H_6) level of theory and thermal correction to free energy obtained at B3LYP-GD3BJ/6-31g(d,p) level of theory.

	**Energy (Hartree)**	**Thermal correction to free energy**
***t*-butylisocyanide**	-250.7920748	0.099856
**Ketenimine**	-752.4573105	0.273012
**Nickelacyclobutane (1)**	-4159.716749	0.679863
**Complex 1-no-P**	-4159.690211	0.672602
**1-CNtBu**	-4410.552554	0.798673
**Complex 2**	-3908.909258	0.615748

**Complex 5**	-4410.549739	0.803883
**Complex 6**	-4410.558108	0.802231
**Complex 6-1**	-4410.547762	0.803358
**Complex 6-eta1(N)**	-4410.55274	0.79577
**Complex 6-eta2(C,N)**	-4410.552268	0.801305
**TS1**	-4410.517709	0.804661
**TS2**	-4410.518345	0.805357
**Complex 7**	-4159.694506	0.67408
**TS3**	-4159.669713	0.674809
**TS4**	-4410.511126	0.799564
**Complex 8**	-4410.51065	0.793516
**TS5**	-4410.499198	0.803017
**TS6**	-4410.498875	0.790617
**1-CntBu-triplet**	-4410.511886	0.795469
**Complex 8-triplet**	-4410.501336	0.792701
**TS7**	-4410.479001	0.792537
**Complex 9**	-4410.524634	0.797332
**Complex 10**	-4410.543317	0.802067
**TS8**	-4410.494113	0.796264
**Complex 11**	-4661.361971	0.927076
**TS9**	-4661.338694	0.926566
**TS 10**	-4661.292184	0.92728
**Complex 11-noP**	-4661.369006	0.925919
**Complex 6-noP**	-4661.361923	0.926449
**TS11**	-4661.294562	0.922954
**Complex 9-triplet**	-4410.528352	0.796116
**Complex 10-triplet**	-4410.510743	0.800071
**TS12**	-4410.515157	0.796355
**TS13**	-4410.501115	0.797073
**Complex 5-triplet**	-4410.523077	0.800923
**Complex 6-1-triplet**	-4410.517961	0.797461
**TS14**	-4410.502704	0.796471
**TS15**	-4410.486605	0.797561
**1-CntBu-no-P**	-4410.53342	0.794703
**Complex 8-no-P**	-4410.487394	0.789699
**TS16**	-4410.466676	0.789752
**Complex 6-no-P**	-4410.536645	0.796096
**TS17**	-4410.461532	0.790765
**Complex 12**	-4410.526096	0.802727

**TS18**	-4410.501141	0.801365
**Complex 13**	-3869.550989	0.589262
**1,1-diphenylethylene**	-540.969905	0.177586
**TS19**	-4410.51466	0.796615
**Complex 1-CO**	-4273.117181	0.682112
**Complex 14**	-4273.103859	0.684989
**TS20**	-4273.081065	0.683962
**Complex 15**	-3732.114589	0.471834
**TS21**	-4273.070179	0.67939
**Complex 16**	-4273.108736	0.683168
**TS22**	-4273.071986	0.684509

6. Literature references

(1) M. L. G. Sansores-Paredes, S. van der Voort, M. Lutz, M.-E. Moret, *Angew. Chem. Int. Ed.* **2021**, *60*, 26518–26522.

(2) M. M. Schreurs, X. Xian, L. M. J. Kroon-Batenburg, *J. Appl. Crystallogr.* **2010**, *43*, 70–82.

(3) R. Herbst-Irmer, G. M. Sheldrick, *Acta Crystallogr. Sect. B Struct. Sci.* **1998**, B*54*, 443–449.

(4) G. G. M. Sheldrick (2014) SADABS and TWINABS. Universität Göttingen, Germany**.**

(5) G. M. Sheldrick, *Acta Crystallogr. Sect. A Found. Crystallogr.* **2015**, A*71*, 3–8.

(6) G. M. Sheldrick, *Acta Crystallogr. Sect. A Found. Crystallogr.* **2015**, C*71*, 3–8.

(7) A. L. Spek, *Acta Crystallogr. Sect. C Struct. Chem.* **2015**, C*71*, 9–18.

(8) A. L. Spek, *Acta Crystallogr. Sect. D Biol. Crystallogr.* **2009**, *65*, 148–155.

(9) M. J. Frisch, G. W. Trucks, H. B. Schlegel, G. E. Scuseria, M. A. Robb, J. R. Cheeseman, G. Scalmani, V. Barone, G. A. Petersson, H. Nakatsuji, X. Li, M. Caricato, A. V. Marenich, J. Bloino, B. G. Janesko, R. Gomperts, B. Mennucci, H. P. Hratchian, J. V. Ortiz, A. F. Izmaylov, J. L. Sonnenberg, D. Williams-Young, F. Ding, F. Lipparini, F. Egidi, J. Goings, B. Peng, A. Petrone, T. Henderson, D. Ranasinghe, V. G. Zakrzewski, J. Gao, N. Rega, G. Zheng, W. Liang, M. Hada, M. Ehara, K. Toyota, R. Fukuda, J. Hasegawa, M. Ishida, T. Nakajima, Y. Honda, O. Kitao, H. Nakai, T. Vreven, K. Throssell, J. A. Montgomery, Jr., J. E. Peralta, F. Ogliaro, M. J. Bearpark, J. J. Heyd, E. N. Brothers, K. N. Kudin, V. N. Staroverov, T. A. Keith, R. Kobayashi, J. Normand, K. Raghavachari, A. P. Rendell, J. C. Burant, S. S. Iyengar, J. Tomasi, M. Cossi, J. M. Millam, M. Klene, C. Adamo, R. Cammi, J. W. Ochterski, R. L. Martin, K. Morokuma, O. Farkas, J. B. Foresman, and D. J. Fox, **2016**, Gaussian 16, Revision C.01, Gaussian, Inc., Wallin.
